# Lung gene expression and single cell analyses reveal two subsets of idiopathic pulmonary fibrosis (IPF) patients associated with different pathogenic mechanisms

**DOI:** 10.1371/journal.pone.0248889

**Published:** 2021-03-23

**Authors:** Jozsef Karman, Jing Wang, Corneliu Bodea, Sherry Cao, Marc C. Levesque

**Affiliations:** Cambridge Research Center, AbbVie, Cambridge, Massachusetts, United States of America; University of Pittsburgh, Dorothy P. and Richard P. Simmons Center for Interstitial Lung Disease, UNITED STATES

## Abstract

Idiopathic pulmonary fibrosis is a progressive and debilitating lung disease with large unmet medical need and few treatment options. We describe an analysis connecting single cell gene expression with bulk gene expression-based subsetting of patient cohorts to identify IPF patient subsets with different underlying pathogenesis and cellular changes. We reproduced earlier findings indicating the existence of two major subsets in IPF and showed that these subsets display different alterations in cellular composition of the lung. We developed classifiers based on the cellular changes in disease to distinguish subsets. Specifically, we showed that one subset of IPF patients had significant increases in gene signature scores for myeloid cells versus a second subset that had significantly increased gene signature scores for ciliated epithelial cells, suggesting a differential pathogenesis among IPF subsets. Ligand-receptor analyses suggested there was a monocyte-macrophage chemoattractant axis (including potentially CCL2-CCR2 and CCL17-CCR4) among the myeloid-enriched IPF subset and a ciliated epithelium-derived chemokine axis (e.g. CCL15) among the ciliated epithelium-enriched IPF subset. We also found that these IPF subsets had differential expression of pirfenidone-responsive genes suggesting that our findings may provide an approach to identify patients with differential responses to pirfenidone and other drugs. We believe this work is an important step towards targeted therapies and biomarkers of response.

## Introduction

Idiopathic pulmonary fibrosis (IPF) is a chronic and progressive fibrosing disease of the lung with a median survival time of <5 years after diagnosis [1, 2]. IPF is characterized histologically by a pattern of usual interstitial pneumonia and the appearance of honeycombing cysts and fibroblastic foci [1, 2]. Although the disease is associated with infiltration and accumulation of inflammatory cells, IPF patients typically do not improve with anti-inflammatory therapy and the only approved IPF therapies, nintedanib and pirfenidone, are anti-fibrotic and not curative [3, 4]. Despite recent advances in genome-wide association studies (GWAS) [5–7], the mechanisms connecting genetic susceptibility, environmental factors and molecular and pathological changes in IPF are incompletely understood.

IPF is a heterogeneous disease with differences in clinical outcome and rates of disease worsening, suggesting that there are subsets of IPF patients with different molecular mechanisms of pathogenesis [8, 9]. As such, a better understanding of IPF pathogenesis and subset heterogeneity is essential to advance new therapies for this devastating disease. A previous attempt by Yang et al. [10] to understand the molecular basis of IPF heterogeneity identified two subsets of IPF patients that were primarily differentiated on the basis of high and low expression of genes from ciliated epithelium; the former was associated with greater pulmonary honeycombing. However, this finding has not been replicated in another study and the pathophysiologic correlates of both subsets have not been explored, including the interactions of ciliated epithelium with other cell types. Cell phenotype-based studies of IPF patient blood and lung samples have shown that increases in plasma cells and mast cells and decreases in T cells were respectively associated with mild versus severe disease [11–13]. Importantly, the overlap between subsets identified using different data sources such as gene expression, and cell phenotypes have not been investigated. The development of molecular classifiers to reliably detect and separate subsets of IPF patients using machine learning has not been attempted.

In the current study, we found that the subsets described by Yang et al. (GSE32537, referred to henceforth as ‘Schwartz-Univ of Colorado bulk expression cohort’) [10] were replicated in our analysis of a new overlapping cohort of IPF patients from a study by Kaminski and colleagues (GSE47460, referred to henceforth as ‘Kaminski-LGRC bulk expression cohort’) [14–17] and in our analysis of a non-overlapping independent cohort of IPF patients (GSE134692 (BMS bulk RNA-seq cohort) [18]). We characterized the cellular changes associated with each subset of IPF patients using cell type signatures derived from recently published single cell RNA sequencing (scRNAseq) data obtained from IPF patients and healthy lungs including GSE132771 (i.e. ‘Sheppard-UCSF single cell cohort’), GSE135893 (‘Kropski-Vanderbilt Univ single cell cohort’) and GSE136831 (‘Kaminski-Yale Univ single cell cohort’) [19, 20, 21]. Importantly, we identified coordinated changes in genes associated with different cell types in each subset of IPF patients that have important implications for the molecular mechanisms driving disease. Finally, we developed molecular classifiers using machine learning approaches to reliably distinguish subsets of patients.

## Methods

### Processing of GSE32537 (Schwartz-Univ of Colorado bulk expression cohort) and GSE47460 (Kaminski-LGRC bulk expression cohort) IPF gene expression dataset

We downloaded and reprocessed microarray data from Schwartz and colleagues (GSE32537, Schwartz-Univ of Colorado bulk expression cohort [10] and Kaminski and colleagues (GSE47460, Kaminski-LGRC bulk expression cohort) [14–17, 22] using ArrayStudio (Qiagen). We applied quantile normalization to the raw data and applied the ‘Remove batch effects’ function in ArrayStudio (Qiagen). We posted normalized gene expression matrices along with the code used to process the data on GitHub (https://github.com/JKarmanAbbVie/IPFproject2020).

### Processing of additional public bulk IPF gene expression studies used in this study

Author-supplied normalized matrix and design files for GSE134692 (BMS bulk RNA-seq cohort) [18] were downloaded from Gene Expression Omnibus. For GSE134692 (BMS bulk RNA-seq cohort) [18], we only used samples from ‘Batch 1’ to avoid batch effects [18]. GSE124685 (‘Kaminski-Yale Univ bulk progression RNA cohort’) [12] RNA sequencing dataset was re-processed from SRA files posted in Gene Expression Omnibus using ArrayStudio. Count data was normalized using the ‘edgeR’ R package [23] (‘TMM’ method) implemented in ArrayStudio. Datasets used in this study are summarized in Table 1.

**Table 1:** Data sets used in this manuscript.

Accession number	Name for dataset used in manuscript	Platform	IPF patients (n)	Healthy controls (n)	References
GSE32537	Schwartz-Univ of Colorado bulk expression cohort	Bulk RNA microarray	119	50	[10]
GSE47460	Kaminski-LGRC bulk expression cohort	Bulk RNA microarray	160	108	[14–17, 22]
GSE124685	Kaminski-Yale Univ bulk progression RNA cohort	Bulk RNA-seq	49	35	[12]
GSE134692	BMS bulk RNA-seq cohort	Bulk RNA-seq	46	26	[18]
GSE132771	Sheppard-UCSF single cell cohort	Single cell RNA-seq	3	3	[19]
GSE135893	Kropski-Vanderbilt Univ single cell cohort	Single cell RNA-seq	19	10	[24]
GSE136831	Kaminski-Yale Univ single cell cohort	Single cell RNA-seq	32	28	[20]

### Identification of gene-expression subsets, principal component analysis and differential gene expression

We used the R package ‘consensusClusterPlus’ [25] to perform unsupervised clustering and identification of subsets based on gene expression using the 5,000 most variable genes (see ‘Code availability’). Performance of consensus clustering was assessed using Proportion of Ambiguous Clustering (PAC) score calculated using R package ‘diceR’ ([26] and https://CRAN.R-project.org/package=diceR). This score has been reported as the best performing metric to assess performance of consensus clustering [23].

Principal component analysis (PCA) on the same 5,000 most variable genes used for consensus clustering was performed using ‘FactoMineR’ and ‘factoextra’ R packages [27, 28]. R package ‘limma’ [29] was used to compare gene expression between subsets of patients. Smoking and gender were included as covariates for linear models in limma. P values were adjusted using the Benjamini-Hochberg FDR procedure and FDR values < 0.05 were considered significant. Upstream regulator analyses were conducted using Ingenuity Pathway Analysis software (Qiagen) with p values and z scores reported (http://pages.ingenuity.com/rs/ingenuity/images/0812%20upstream_regulator_analysis_whitepaper.pdf). KEGG, Gene Ontology and Reactome pathway analyses were performed using ‘clusterProfiler’ and ‘ReactomePA’ R packages [30, 31].

### Processing of single cell RNA datasets, development of cell signature scores and application of cell signature scores to GSE47460 (Kaminski-LGRC bulk expression cohort) and GSE134692 (BMS bulk RNA-seq cohort)

Single cell data from Tsukui et al. (GSE132771, Sheppard-UCSF single cell cohort) [19], Habermann et al. (GSE135893, Kropski-Vanderbilt Univ single cell cohort) [24] and Adams et al. GSE136831 (Kaminski-Yale Univ single cell cohort) [20] were either processed with filtered sparse matrix output from ‘cellranger’ (10x Genomics) (GSE132771 (Sheppard-UCSF single cell cohort) [19], GSE136831 (Kaminski-Yale Univ single cell cohort) [20] or we used the analyzed data provided by the authors (GSE135893, Kropski-Vanderbilt Univ single cell cohort) [24]. Sparse matrices were processed using the R package ‘Seurat’ [32]. Cell cluster signatures were determined using differential gene expression with the R package ‘MAST’ (https://github.com/RGLab/MAST/) and by maximizing the power of the gene signature to discriminate a particular cell type from the other cell types using an AUROC-based metric (see ‘Code availability’ and reference [33]). To summarize, we first annotated cell clusters in the scRNAseq data based on canonical markers. We calculated differentially expressed genes (DEGs) for each cluster by comparing the cluster to all other cells in the dataset using the ‘FindMarkers’ function in the R package ‘Seurat’ [32]. We then ranked DEGs in decreasing order according to their effect sizes and performed a step-wise search to identify the smallest gene signature that accurately classified a given cell type from every other cell type in the scRNAseq dataset [33]. This included the following steps: (1) estimation of the classification accuracy of the first gene on the list using the area under receiver operating characteristic (AUROC) curve; (2) incremental addition of one gene at a time based on the gene’s rank and re-computation of the AUROC corresponding to the new gene set, and 3.) repetition of this process until we identified the minimal gene set that produced an AUROC proximal to the maximum (with ε = 0.005), requiring a minimum of 5 genes per signature. We performed this strategy on each cell type across the scRNAseq dataset [33]. This method focused on finding the best performing gene set that distinguished a given cell type from the rest of the cell types in the dataset and therefore resulted in cell type signatures with partial overlaps with gene signatures from other cell types in the dataset.

We created two sets of gene expression signatures for GSE132771 (Sheppard-UCSF single cell cohort) [19]: one for total lung cell suspension samples (sample identifiers GSM3891621, GSM3891623, GSM38916215, GSM3891627, GSM3891629, GSM3891631) and one for ‘Lineage-sorted samples’ (GSM3891620, GSM3891622, GSM3891624, GSM3891626, GSM3891628, GSM3891630). These two separate sets of gene signatures were created to achieve better resolution of mesenchymal cell types as previously described [19]. We created one set of gene expression signatures each for GSE135893 (Kropski-Vanderbilt Univ single cell cohort) [24] as this dataset included only total lung suspension samples. We calculated correlation matrices for each signature score derived from GSE47460 so its performance could be assessed (S3 and S5 Figs) using R package ‘ggcorrplot’ (https://github.com/kassambara/ggcorrplot).

Gene signature scores from bulk IPF RNA microarray (GSE47460, Kaminski-LGRC bulk expression cohort, [14–17, 22]) and RNA-seq (GSE134692, BMS bulk RNA-seq cohort, [18]) were calculated using normalized, batch-corrected gene expression data using the ‘GSVA’ R package with ‘method = ‘gsva” setting to calculate gene signature enrichment scores using gene sets derived from the single cell RNA sequencing data. We used the Gene Set Variation Analysis (GSVA) method as described [34]. The GSVA method has several advantages over previously published gene set enrichment methods such as combined z-score, PLAGE and ssGSEA [35–37] since GSVA calculated sample-wise gene enrichment scores as a function of genes inside and outside the gene set specified and estimated variation of gene set enrichment over samples independent of any class label in a non-parametric, unsupervised manner [34]. GSVA also alleviated the issue of partially overlapping signatures in the cell type signatures as it relies on the ranking of the entire gene set used as input and used efficient normalization and outlier removal methods so a given cell type-specific signature was not driven by outlying genes but was driven instead by the entire signature [34]. We used changes in gene signature scores to estimate changes in cell type composition in bulk RNA microarray and RNA-seq data.

GSVA-derived gene signature scores from GSE47460 (Kaminski-LGRC bulk expression cohort) [14–17, 22] and RNA-seq (GSE134692, BMS bulk RNA-seq cohort, [18]) were compared between subsets of IPF patients and controls using one-way analysis of variance (ANOVA) followed by non-parametric Dunn’s post-hoc test with the null hypothesis of the groups being not different. Benjamini-Hochberg-adjusted p values < 0.05 were considered significant.

### Determination of ligand-receptor interactions using single cell RNA datasets

We calculated cell type percentages in GSE135893 (Kropski-Vanderbilt Univ single cell cohort) [24] by dividing the number of cells in each cluster by the total number of cells in the data for the purposes of creating patient subsets in GSE135893 [24]. Subsequently, ligand-receptor interactions from single cell RNA sequencing data were inferred using the PyMINEr [38] and NicheNet [39] R packages. PyMINEr was implemented as an R package and used ligand-receptor network inference from single cell data (Clarivate Analytics; Philadelphia, PA). Ligand-receptor interactions were obtained from Ramilowski et al. [40]. Chord diagrams were created using the R package ‘circlize’ [41].

### Development of classifiers for subsets in GSE47460 (Kaminski-LGRC bulk expression cohort)

The R package ‘caret’ (https://github.com/topepo/caret/) was used to build classifiers and feature selection from the GSE47460 (Kaminski-LGRC bulk expression cohort) dataset [14–17]. We used subsets 1 and 2 of the IPF patients as the outcome and built models using ‘svmLinear’, ‘gbm’ and ‘glmnet’ in caret (see ‘Code availability’). Performance of the models was evaluated using the ‘MLeval’ R package.

### Development of pirfenidone response signature

We used the combination of genes significantly downregulated by pirfenidone as reported in Supplementary Table 1 of reference [42]. We combined genes downregulated in response to pirfenidone in lung homogenates only (labeled ‘LH only’ in [42]) and downregulated in both lung homogenates and isolated fibroblasts (labeled ‘both down’ in [42]) using a log fold change cutoff of 1.41 and p value cutoff of 0.05. Pirfenidone signature score was calculated using the GSVA method as outlined above for scRNAseq signature scores.

## Results

### Consensus clustering results using data from GSE47460 (Kaminski-LGRC bulk expression cohort)

**Fig 1:**
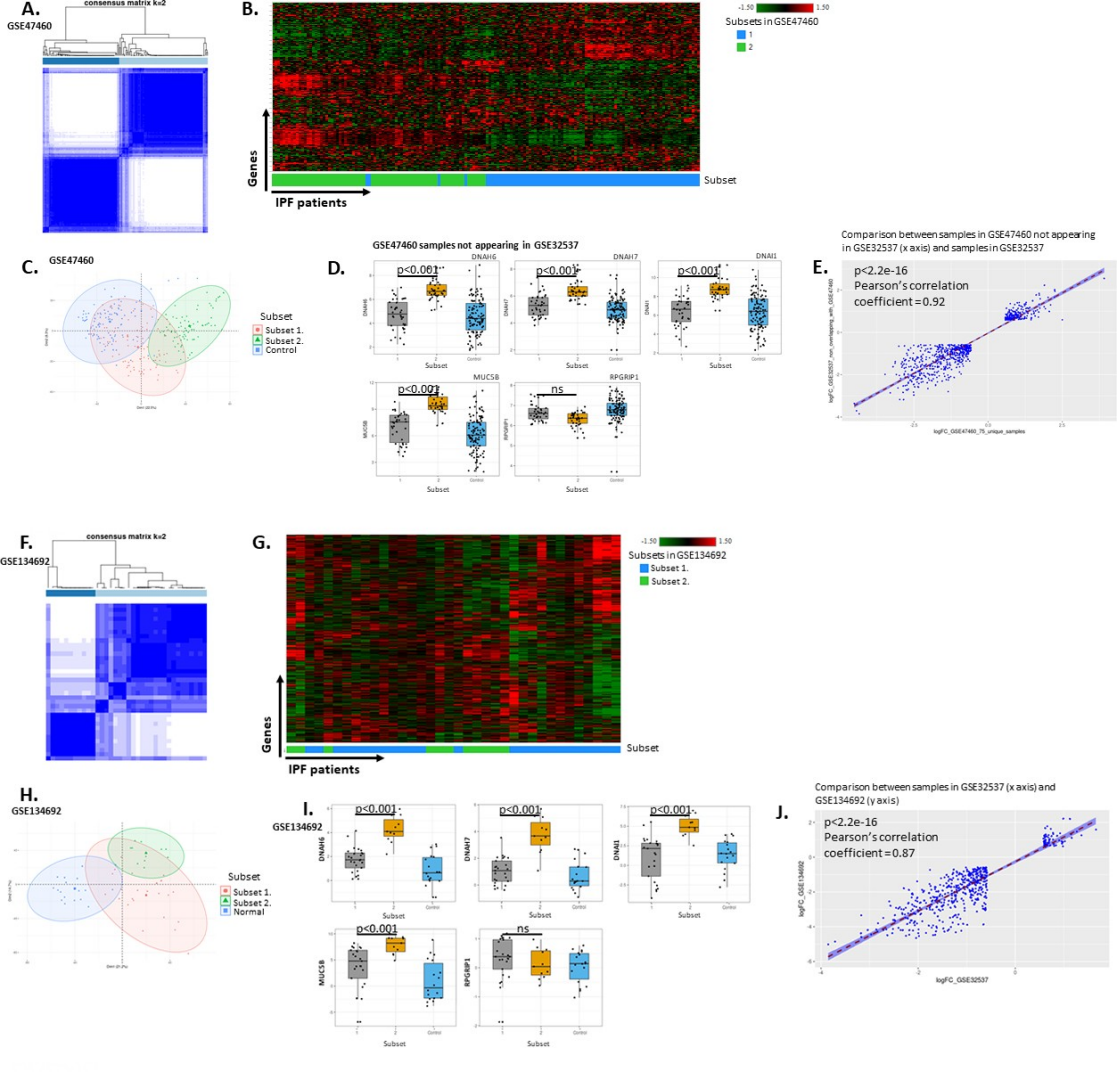
Consensus clustering results of cohort GSE47460 (Kaminski-LGRC bulk expression cohort) [14–17], GSE134692 (BMS bulk RNA-seq cohort) [18] and replication of GSE32537 (Schwartz-Univ of Colorado bulk expression cohort) [10] results. A. Consensus clustering of IPF patients in GSE47460 (Kaminski-LGRC bulk expression cohort) [14–17] based on the 5,000 most variable genes in IPF patients showing distribution of samples based on k = 2 consensus clusters. B. Hierarchical clustering of IPF samples from GSE47460 (Kaminski-LGRC bulk expression cohort) [14–17] using top 5,000 most variable genes. x axis represents individual patients, y axis represents genes. Subsets are indicated in x axis color bar and legend of heatmap and correspond to classes shown in Fig 1A. C. PCA of IPF and Control samples from GSE47460 (Kaminski-LGRC bulk expression cohort) [14–17] with IPF subsets and Control indicated. Subsets are indicated in legend of PCA plot and correspond to classes shown in Fig 1A. D. Expression of cilium-related genes previously identified by Yang et al. in [10] from the 75 samples in GSE47460 (Kaminski-LGRC bulk expression cohort) [14–17] not overlapping with GSE32537. Subsets are indicated on x axis of box plots and correspond to classes shown in Fig 1A. Adjusted p values determined by ANOVA and post-hoc Dunn’s test are reported on plots. E. Correlation plot of log fold changes calculated in GSE47460 (Kaminski-LGRC bulk expression cohort) [14–17] (when comparing Subset 1 and Subset 2 (x axis) using the 75 samples not appearing in GSE32537 and compared to GSE32537 (Schwartz-Univ of Colorado bulk expression cohort) [10] (y axis). Genes with reported absolute log fold change of larger than 0.58 and adjusted p value < 0.05 were used in this analysis from both datasets. F. Consensus clustering of IPF patients in GSE134692 (BMS bulk RNA-seq cohort) [18] based on the 5,000 most variable genes in IPF patients showing distribution of samples based on k = 2 consensus clusters. G. Hierarchical clustering of IPF samples from GSE134692 (BMS bulk RNA-seq cohort) [18] using top 5,000 most variable genes. x axis represents individual patients, y axis represents genes. Subsets are indicated in x axis color bar and legend of heatmap and correspond to classes shown in Fig 1F. H. PCA of IPF and Normal samples from GSE134692 (BMS bulk RNA-seq cohort) [18] with IPF subsets and Normal indicated. Subsets are indicated in legend of PCA plot and correspond to classes shown in Fig 1F. I. Expression of cilium-related genes from GSE134692 (BMS bulk RNA-seq cohort) [18] previously identified by Yang et al. [10]. Subsets are indicated on x axis of box plots and correspond to classes shown in Fig 1F. Adjusted p values determined by ANOVA and post-hoc Dunn’s test are reported on plots. J. Correlation plot of log fold changes calculated in GSE32537 (Schwartz-Univ of Colorado bulk expression cohort) [10] (when comparing Subset 1 and Subset 2 (x axis, logFC_GSE32537) compared to GSE134692 (BMS bulk RNA-seq cohort) [18] subsets (y axis, logFC_GSE134692). Genes with reported absolute log fold change of larger than 0.58 and adjusted p value < 0.05 were used in this analysis from both datasets.

A previous attempt to identify subsets of IPF patients based on total lung gene expression identified subsets with large differences in cilia-related gene expression and MUC5B gene expression levels (GSE32537, Schwartz-Univ of Colorado bulk expression cohort) [10]. Therefore, we initially repeated and confirmed the identification of the same two IPF patient subsets in GSE32537 (Schwartz-Univ of Colorado bulk expression cohort) [10] and used GSE47460 (Kaminski-LGRC bulk expression cohort) [14–17, 22] as a replication cohort. We applied the same consensus clustering approach on both datasets for the sake of consistency in processing the data instead of the subsetting method used in the original publication of GSE32537 (Schwartz-Univ of Colorado bulk expression cohort) [10]. Importantly, this approach had the advantage that it analyzed a partially independent patient cohort (see below; GSE47460, Kaminski-LGRC bulk expression cohort) [14–17] that measured gene expression on a platform (Agilent) different from that used in the original GSE32537 (Schwartz-Univ of Colorado bulk expression cohort) study (Affymetrix 1.0ST) [10]. We used a data-driven, hypothesis-free approach of consensus clustering of the data obtained from IPF patients in GSE47460 (Kaminski-LGRC bulk expression cohort) [14–17], and elected to use k = 2 as the number of consensus clusters (‘consensusclasses’) in both the GSE32537 (Schwartz-Univ of Colorado bulk expression cohort) [10] and the GSE47460 (Kaminski-LGRC bulk expression cohort) [14–17] based on the consensus clustering results (Fig 1A and S1 Fig). We calculated the Proportion of Ambiguous Clustering (PAC) score [26] to assess performance of the consensus clustering process for a range of possible cluster numbers. PAC scores are regarded as the best current metric for assessing clustering performance (the lower, the better performance of clustering) [26]. In our consensus clustering results of GSE32537 (Schwartz-Univ of Colorado bulk expression cohort) [10], k = 2 produced the highest PAC scores. In our consensus clustering analysis of GSE47460 (Kaminski-LGRC bulk expression cohort) [14–17], the PAC score was lower for k = 2 than for k = 3 or k = 4 and minimally higher than k = 5 (S1A Fig). We elected to use k = 2 clusters for subsequent analyses to balance good performance of clustering and reasonable sample numbers for achieving adequate statistical power. With k = 2 clusters, 53% of patients were in consensus class 1 and 47% of patients were in consensus class 2 in GSE47460 (Kaminski-LGRC bulk expression cohort) [14–17] (Fig 1A), thereby presenting a well-balanced dataset. Hierarchical clustering based on the 5,000 most variable genes in GSE47460 (Kaminski-LGRC bulk expression cohort) [14–17] showed the distribution and relative gene expression of the two IPF subsets (Fig 1B). We will subsequently refer to consensus class 1 as ‘Subset 1’ and consensus class 2 as ‘Subset 2’.

We performed a PCA of the GSE47460 (Kaminski-LGRC bulk expression cohort) [14–17] dataset to identify the main features contributing to the separation of the subsets. We included normal control samples in the PCA to understand how the two subsets of IPF patients separated from each other and from healthy control samples. As shown in Fig 1C, the first principal component had the strongest association with the subject cohort (IPF patient or healthy subject). The PCA analysis of IPF samples indicated that there was no correlation with any of the clinical characteristics reported by the authors. We used differentially expressed genes in Subset 1 and Subset 2 as compared to healthy controls to conduct pathway enrichment using the Ingenuity Pathway Analysis tool. With the same data, we conducted a gene set enrichment analysis using the Reactome pathway database (Table 2). Both Subset 1 and 2 showed an enrichment in extracellular matrix-related processes (Table 2). Importantly, in IPA analyses, only Subset 1 showed an enrichment in ‘Role of Macrophages Fibroblasts and Endothelial Cells in Rheumatoid Arthritis’ and only Subset 2 showed an enrichment in cilium biology-related Reactome pathways (Table 2).

**Table 2:** Pathway enrichment results in patient subsets in GSE47460 (Kaminski-LGRC bulk expression cohort) [14–17].

**A. Top Enriched IPA Pathways in Subset 1**									**B. Top Enriched IPA Pathways in Subset 2**								
**Ingenuity Canonical Pathways**							**-log(p-value)**		**Ingenuity Canonical Pathways**						**-log(p-value)**		
Hepatic Fibrosis / Hepatic Stellate Cell Activation							19.9		Granulocyte Adhesion and Diapedesis						17.4		
Granulocyte Adhesion and Diapedesis							15.4		Hepatic Fibrosis / Hepatic Stellate Cell Activation						14.1		
Agranulocyte Adhesion and Diapedesis							13.5		Agranulocyte Adhesion and Diapedesis						13.7		
Osteoarthritis Pathway							11.4		Atherosclerosis Signaling						10.1		
Axonal Guidance Signaling							8.83		Breast Cancer Regulation by Stathmin1						10.1		
Airway Pathology in Chronic Obstructive Pulmonary Disease							8.16		Osteoarthritis Pathway						9.64		
Role of Osteoblasts, Osteoclasts and Chondrocytes in Rheumatoid Arthritis							8.03		Axonal Guidance Signaling						9.52		
Role of Macrophages, Fibroblasts and Endothelial Cells in Rheumatoid Arthritis							7.65		LXR/RXR Activation						8.78		
Breast Cancer Regulation by Stathmin1							7.37		Role of Osteoblasts, Osteoclasts and Chondrocytes in Rheumatoid Arthritis						7.56		
Inhibition of Matrix Metalloproteases							7.14		cAMP-mediated signaling						6.92		
**C. Top Activated Reactome Pathways in Subset 1**									**D. Top Activated Reactome Pathways in Subset 2**								
**Pathway**	**Description**	**set Size**	**enrichment Score**	**NES**	**p value**	**adjusted p value**		**q value**	**Pathway**	**Description**	**set Size**	**enrichment Score**	**NES**	**p value**		**adjusted p value**	**q value**
R-HSA-1442490	Collagen degradation	29	0.6439	2.88	0.0001	0.0019		0.0013	R-HSA-6805567	Keratinization	42	0.6099	2.52	0.0001		0.0064	0.0050
R-HSA-1474228	Degradation of the extracellular matrix	47	0.5509	2.86	0.0001	0.0019		0.0013	R-HSA-6809371	Formation of the cornified envelope	42	0.6099	2.52	0.0001		0.0064	0.0050
R-HSA-1474290	Collagen formation	28	0.6240	2.76	0.0001	0.0019		0.0013	R-HSA-1474228	Degradation of the extracellular matrix	54	0.5163	2.26	0.0001		0.0064	0.0050
R-HSA-2022090	Assembly of collagen fibrils and other multimeric structures	22	0.6699	2.75	0.0002	0.0019		0.0013	R-HSA-1442490	Collagen degradation	34	0.5622	2.21	0.0001		0.0064	0.0050
R-HSA-1474244	Extracellular matrix organization	78	0.4502	2.64	0.0001	0.0019		0.0013	R-HSA-1592389	Activation of Matrix Metalloproteinases	19	0.6468	2.19	0.0001		0.0064	0.0050
R-HSA-1650814	Collagen biosynthesis and modifying enzymes	23	0.6172	2.57	0.0002	0.0019		0.0013	R-HSA-5617833	Cilium Assembly	50	0.4519	1.95	0.0014		0.0360	0.0284
R-HSA-8948216	Collagen chain trimerization	18	0.6468	2.47	0.0003	0.0030		0.0021	R-HSA-1474290	Collagen formation	37	0.4759	1.91	0.0026		0.0432	0.0341
R-HSA-6805567	Keratinization	19	0.6327	2.47	0.0002	0.0019		0.0013	R-HSA-1852241	Organelle biogenesis and maintenance	53	0.4238	1.85	0.0028		0.0432	0.0341
R-HSA-6809371	Formation of the cornified envelope	19	0.6327	2.47	0.0002	0.0019		0.0013	R-HSA-2022090	Assembly of collagen fibrils and other multimeric structures	28	0.4891	1.84	0.0049		0.0515	0.0406
R-HSA-1640170	Cell Cycle	51	0.4204	2.23	0.0003	0.0029		0.0020	R-HSA-69620	Cell Cycle Checkpoints	46	0.4288	1.81	0.0055		0.0515	0.0406

As the set of samples between GSE32537 (Schwartz-Univ of Colorado bulk expression cohort) [10] and GSE47460 (Kaminski-LGRC bulk expression cohort) [14–17] are partially overlapping, we further validated our findings from the GSE47460 (Kaminski-LGRC bulk expression cohort) [14–17] dataset. We approached this in two different ways: (1) by separately analyzing samples different between GSE32537 (Schwartz-Univ of Colorado bulk expression cohort) [10] and GSE47460 (Kaminski-LGRC bulk expression cohort) [14–17] datasets; and by (2) analyzing a completely independent cohort of IPF patients (GSE134692 (BMS bulk RNA-seq cohort) [18]).

For the first approach, we used Gene Expression Omnibus records to analyze both non-overlapping IPF data from GSE32537 (Schwartz-Univ of Colorado bulk expression cohort) [10] and GSE47460 (Kaminski-LGRC bulk expression cohort) [14–17]. 85 out of 160 IPF subjects in GSE47460 (Kaminski-LGRC bulk expression cohort) [14–17] overlapped with GSE32537 (Schwartz-Univ of Colorado bulk expression cohort) [10] and 75 samples were unique to the GSE47460 Kaminski-LGRC bulk expression cohort (please see ‘Code availability’). Therefore, we performed the same Consensus Clustering analysis on the set of 75 non-overlapping samples between GSE47460 (Kaminski-LGRC bulk expression cohort) [14–17] and GSE32537 (Schwartz-Univ of Colorado bulk expression cohort) [10] that we used on the entire GSE47460 (Kaminski-LGRC bulk expression cohort) [14–17] dataset of 160 samples. The unique set of 75 samples in GSE47460 (Kaminski-LGRC bulk expression cohort) [14–17] not overlapping with GSE32537 (Schwartz-Univ of Colorado bulk expression cohort) [10] showed a similar pattern of consensus clustering of two subsets (S1C and S1D Fig). There was no significant skewing relative to the entire dataset of 160 samples in either the overlapping set of 85 samples or the non-overlapping set of 75 samples between GSE47460 (Kaminski-LGRC bulk expression cohort) [14–17] and GSE32537 (Schwartz-Univ of Colorado bulk expression cohort) [10] (S1C Fig; p = 0.64).

We next determined whether the subsets identified by consensus clustering in GSE47460 (Kaminski-LGRC bulk expression cohort) [14–17] overlapped with the subsets reported by Yang et al. (GSE32537, Schwartz-Univ of Colorado bulk expression cohort) [10] using expression of the same set of 5 genes (RPGRIP1, DNAH6, DNAH7, DNAI1, MUC5B) reported by Yang et al. as significantly different between subsets in GSE32537 (Schwartz-Univ of Colorado bulk expression cohort) [10]. As shown in Fig 1D, the two subsets identified in our study of the 75 samples in GSE47460 (Kaminski-LGRC bulk expression cohort) [14–17] not overlapping with GSE32537 (Schwartz-Univ of Colorado bulk expression cohort) [10] showed a very similar pattern of expression of these 5 genes with evidence of elevated expression of ciliated epithelium-related genes in Subset 2. An analysis of the correlation between changes in gene expression between IPF patient subsets in GSE32537 (Schwartz-Univ of Colorado bulk expression cohort) [10] and the gene expression changes between IPF patient subsets in the 75 unique (not overlapping with GSE32537 (Schwartz-Univ of Colorado bulk expression cohort) [10]) samples in GSE47460 (Kaminski-LGRC bulk expression cohort) [14–17] showed a high level of correlation, indicating the reproducibility of subsets in the GSE32537 (Schwartz-Univ of Colorado bulk expression cohort) [10] cohort and the unique (not overlapping with GSE32537 (Schwartz-Univ of Colorado bulk expression cohort) [10]) samples in GSE47460 (Kaminski-LGRC bulk expression cohort) [14–17] (Fig 1E). We also reproduced an upstream regulator analysis from the Yang et al. study (GSE32537, Schwartz-Univ of Colorado bulk expression cohort) [10] using data from the GSE47460 (Kaminski-LGRC bulk expression cohort) [14–17] study and found that Subsets 1 and 2 had similar upstream regulators in both the GSE32537 (Schwartz-Univ of Colorado bulk expression cohort) [10] and GSE47460 (Kaminski-LGRC bulk expression cohort) [14–17] datasets. Altogether, this analysis indicated that the two subsets identified in GSE47460 (Kaminski-LGRC bulk expression cohort) [14–17] IPF patients significantly overlapped with the same two subsets of IPF patients identified in GSE32537 (Schwartz-Univ of Colorado bulk expression cohort) [10].

To further substantiate our results, we analyzed an additional independent cohort of IPF patients (GSE134692 (BMS bulk RNA-seq cohort) [18] to validate our findings. To the best of our knowledge, GSE134692 (BMS bulk RNA-seq cohort) [18] used a completely non-overlapping set of samples with LGRC. Consensus clustering results using GSE134692 (BMS bulk RNA-seq cohort) [18] also revealed two main subsets of IPF patients (Fig 1F–1J and S1B Fig). We performed a correlation analysis between GSE134692 (BMS bulk RNA-seq cohort) [18] and GSE32537 (Schwartz-Univ of Colorado bulk expression cohort) [10]. This analysis showed a high level of correlation similar to that detected between GSE32537 (Schwartz-Univ of Colorado bulk expression cohort) [10] and GSE47460 (Kaminski-LGRC bulk expression cohort) [14–17] (Fig 1J). Therefore, the two main subsets of IPF patients detected originally in GSE32537 (Schwartz-Univ of Colorado bulk expression cohort) [10] were reproduced across both partially overlapping (GSE47460 (Kaminski-LGRC bulk expression cohort) [14–17]) and completely independent (GSE134692 (BMS bulk RNA-seq cohort) [18]) patient cohorts and across technology platforms.

### Markers of fibrosis and differences in clinical data in IPF subsets

**Fig 2:**
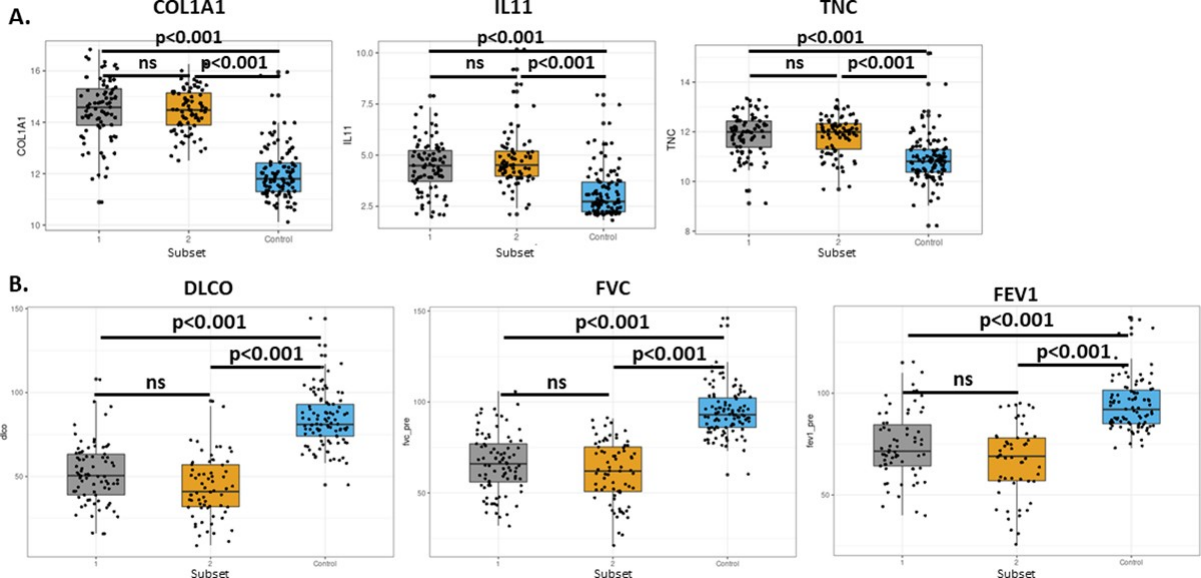
Evaluation of fibrotic markers and clinical parameters in IPF subsets. A. Expression of fibrosis markers in IPF subsets based on the analysis in Fig 1 in GSE47460 (Kaminski-LGRC bulk expression cohort) [14–17] as compared to healthy controls (‘Control’). Adjusted p values determined by ANOVA and post-hoc Dunn’s test are reported on plots. B. Distribution of clinical parameters in IPF subsets based on the analysis in Fig 1 as reported in GSE47460 (Kaminski-LGRC bulk expression cohort) [14–17] as compared to healthy controls (‘Control’). %D_LCO_, FVC and FEV1 values represent pre-lung transplant values reported in GSE47460 (Kaminski-LGRC bulk expression cohort) [14–17]. Adjusted p values determined by ANOVA and post-hoc Dunn’s test are reported on plots.

We next determined whether markers of fibrosis and clinical data associated with the severity of IPF were different between the two subsets identified in GSE47460 (Kaminski-LGRC bulk expression cohort) [14–17]. We did not detect changes in the expression of fibrotic genes (including collagen expression, tenascin C (TNC) and IL-11 mRNA levels) between Subset 1 and 2 in GSE47460 (Kaminski-LGRC bulk expression cohort) [14–17] (Fig 2A); these results were consistent with results from the study by Yang et al. (GSE32537, Schwartz-Univ of Colorado bulk expression cohort) [10]. We found no significant differences in percent diffusing capacity of carbon monoxide (%DlCO), forced expiratory volume in 1 second (FEV1) or forced vital capacity (FVC) between the two subsets of patients in GSE47460 (Kaminski-LGRC bulk expression cohort) [14–17] (Fig 2B); these findings were mostly consistent with the reported differences in honeycombing only in IPF patients in the study by Yang et al. (GSE32537, Schwartz-Univ of Colorado bulk expression cohort) [10], in which patients with prominent ciliated epithelial gene expression had worse honeycombing. Overall, this indicated that analysis of clinical parameters, by including additional samples from GSE47460 (Kaminski-LGRC bulk expression cohort) [14–17], did not change the original findings and conclusions from GSE32537 (Schwartz-Univ of Colorado bulk expression cohort) [10] related to the IPF patient subsets. Interestingly, 3 out of the 4 markers (*GREM1*, *MMP7*, *CTHRC1* and *FHL2*) identified by Kaminski and colleagues [43] as having a significant negative correlation with %DlCO and as markers that separate IPF patients by disease severity were expressed at a higher level in Subset 2 of GSE47460 (Kaminski-LGRC bulk expression cohort) [14–17] (S2 Fig), which suggested a differential prognosis for the two subsets of IPF patients. *MMP7* and *FHL2* were also reported to be different between subsets in the study by Yang et al. (GSE32537, Schwartz-Univ of Colorado bulk expression cohort) [10]. We did not detect differences in age, sex and smoking history between Subset 1 and 2, which is in agreement with the earlier findings by Yang et al. (GSE32537, Schwartz-Univ of Colorado bulk expression cohort) [10].

### Cellular changes in IPF subsets

The pathological process in IPF leads to marked changes in cellular composition of lung tissue and is associated with alterations in both hematopoietic and non-hematopoietic cell populations [44]. Advances in single cell RNA sequencing (scRNAseq) have enabled the quantification of these cellular changes at an unprecedented resolution. To this end, we developed a pipeline based on differential expression of genes in clusters of cells identified in IPF lung samples and created gene signatures from recently published scRNAseq data [9–12]. This approach bridged the problems inherent to low sample sizes in scRNAseq datasets that precluded reliable consensus clustering. For this analysis, we first used scRNAseq results from Tsukui et al. (GSE132771, Sheppard-UCSF single cell cohort) [19] to develop cellular signatures by re-analyzing the raw data from GSE132771 (Sheppard-UCSF single cell cohort) [19]. Clusters identified through the reanalysis of published scRNAseq data matched well with the clusters published by Tsukui et al. (S3 Fig). We determined cell type-specific gene sets using the methods described in [33]. Cell signatures for each cell type are listed in S1 Table.

We applied the cellular signatures derived from Tsukui et al. GSE132771 (Sheppard-UCSF single cell cohort) [19] to the data from Kaminski and colleagues (GSE47460, Kaminski-LGRC bulk expression cohort) [14–17]. First, we assessed the overlap of genes and the correlation of gene signature scores derived from GSE47460 (Kaminski-LGRC bulk expression cohort) [14–17]) (S3 and S5 Figs). This assessment showed the expected low level of correlation of scores between cells of mesenchymal and hematopoietic origin (S3C and S5B Figs). The highest correlation coefficient observed across the mesenchymal cell populations in the total lung cell suspension dataset (S5A Fig) was less than 0.5 (S3C Fig). In the ‘Lineage sorted’ dataset (S3B Fig), higher correlation coefficients were observed as these cell types are expected to be developmentally and functionally more similar (S3D Fig).

**Fig 3:**
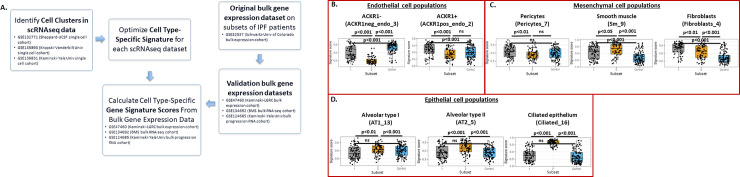
Gene signature scores for non-hematopoietic cell populations in GSE47460 (Kaminski-LGRC bulk expression cohort) [14–17] subclasses. Signatures were determined using total lung mononuclear cell data from GSE132771 (Sheppard-UCSF single cell cohort) [19]. Cell cluster names follow labeling in S3A Fig. Labels used in S3 Fig are indicated in parentheses. A. Details of our workflow with datasets used at each step indicated. B. Gene signature scores for endothelial cell subpopulations. C. Gene signature scores for mesothelial cell populations. D. Gene signature scores for epithelial cell subpopulations. Adjusted p values are reported on plots.

**Fig 4:**
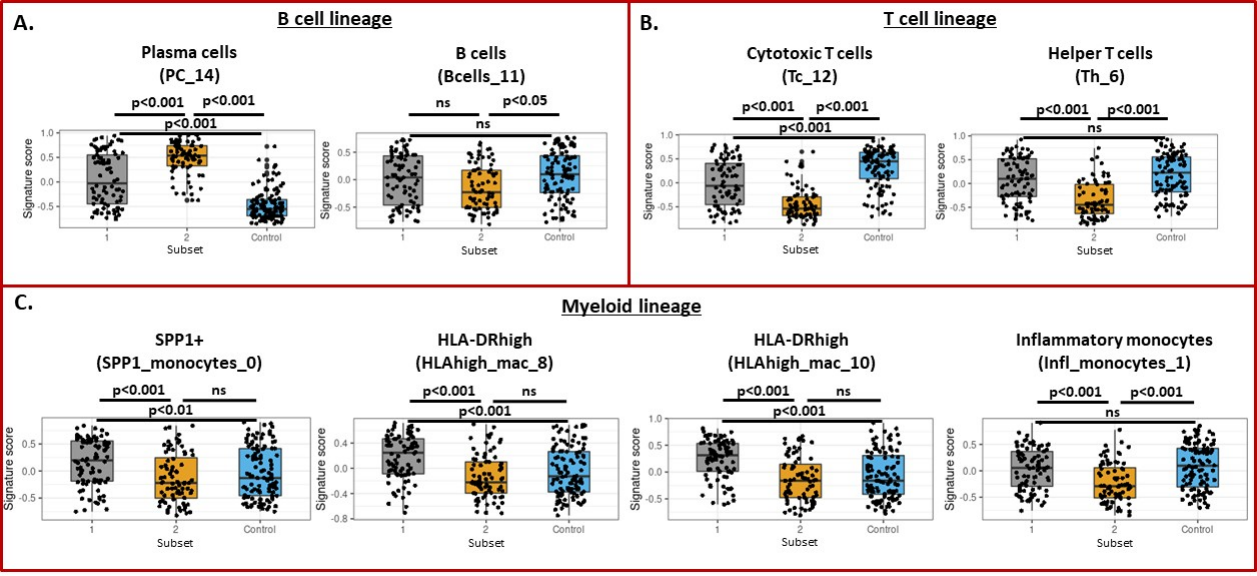
Gene signature scores for hematopoietic cell populations in GSE47460 (Kaminski-LGRC bulk expression cohort) [14–17] subclasses. Signatures were determined using total lung mononuclear cell data from GSE132771 (Sheppard-UCSF single cell cohort) [19]. Cell cluster names follow labeling in S3A Fig. Labels used in S3A Fig are indicated in parentheses. A. Gene signature scores for B cell subpopulations. B. Gene signature scores for T cell populations. C. Gene signature scores for myeloid cell subpopulations. Adjusted p values are reported on plots.

Next, we calculated gene signature scores using Gene Set Variation Analysis (GSVA) as described in ‘Methods’ and observed strong, coordinated changes in gene signature scores for epithelial and endothelial cell populations that differed significantly between Subsets 1 and 2 (Fig 3). Our workflow is detailed in Fig 3A. We detected the most significant differences in gene signature scores for ACKR1 negative endothelial cells and ciliated epithelial cells (Fig 3B and 3D). The latter result matched well with the finding that ciliated epithelium-related gene expression was significantly higher in Subset 2 of IPF patients as previously shown in Fig 1D. Among hematopoietic cell populations, gene expression scores also differed significantly between Subsets 1 and 2 (Fig 4). Specifically, Subset 2 had higher levels of B cells/ plasma cells and lower levels of T cells compared to Subset 1 (Fig 4A and 4B). Gene signature scores for monocytes and macrophages were increased in Subset 1 versus Subset 2 (Fig 4C). This conclusion was also supported by the expression of individual marker genes across patient subsets (examples are shown in S4 Fig).

To confirm the findings using cellular signatures developed from the data of Tsukui et al. (GSE132771, Sheppard-UCSF single cell cohort) [19], we used more recent scRNAseq datasets from Habermann et al. (GSE135893, Kropski-Vanderbilt Univ single cell cohort) [24] to repeat the development of cellular signatures (S5 Fig). Cellular signatures developed using GSE135893 (Kropski-Vanderbilt Univ single cell cohort) [24] showed similar overlap and correlation structure to GSE132771 (Sheppard-UCSF single cell cohort, S5B and S3C Figs). Using gene signatures from GSE135893 (Kropski-Vanderbilt Univ single cell cohort), we replicated the results from the signatures developed from Tsukui et al. (GSE132771, Sheppard-UCSF single cell cohort) [19] and also identified an increase in mast cells in Subset 1 (S6 Fig).

Additionally, to further validate this approach in additional IPF cohorts with bulk transcriptomic data, we repeated consensus clustering and cell type signature analysis of an additional IPF cohort in which bulk RNAseq data was available (from lungs removed from IPF and control patients, GSE134692 (BMS bulk RNA-seq cohort)) [18]. This analysis showed similar trends to those seen in GSE47460 (Kaminski-LGRC bulk expression cohort) [14–17]; there were two main subsets of patients in GSE134692 (BMS bulk RNA-seq cohort) with one subset expressing high levels of ciliated epithelium-related genes and another subset enriched in macrophage gene signatures (S7 Fig).

### Changes in fibroblast, pericyte and smooth muscle populations in IPF subsets

**Fig 5:**
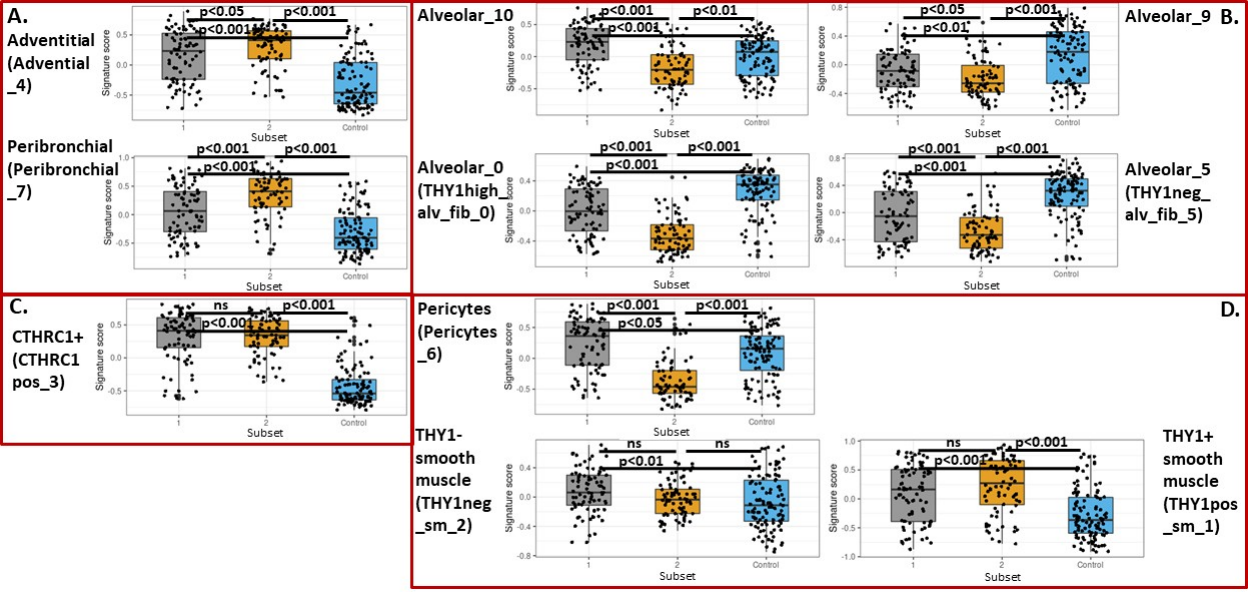
Gene signature scores for smooth muscle/pericyte/fibroblast obtained by using CD45-/EPCAM-/CD235a- (‘Lineage-sorted cells’) data from GSE132771 (Sheppard-UCSF single cell cohort) [19] in GSE47460 (Kaminski-LGRC bulk expression cohort) [14–17] subclasses. Cell cluster names follow labeling in S3B Fig. Labels used in S3B Fig are indicated in parentheses. A. Gene signature scores for adventitial and peribronchial fibroblast subpopulations. B. Gene signature scores for alveolar fibroblast subpopulations. C. Gene signature scores for CTHRC1^+^ fibroblast subpopulation. D. Gene expression scores for pericytes and smooth muscle cell populations. Adjusted p values are reported on plots.

The scRNAseq data allowed identification of novel subtypes of fibroblasts in IPF as reported by Tsukui et al. [19]. Importantly, these authors identified a subset of disease-specific fibroblasts in IPF characterized by high level expression of the CTHRC1 gene and pro-fibrotic mediators including type I and III collagen. Using the gene expression signatures and the same methods, we evaluated changes in fibroblast subpopulations in the two subsets identified in GSE47460 (Kaminski-LGRC bulk expression cohort) [14–17] (Fig 5). Gene signature scores for many of the fibroblast, pericyte and smooth muscle subpopulations were similarly enriched in IPF patient Subsets 1 and 2. However, gene signature scores for alveolar fibroblast populations were more enriched in Subset 1, whereas gene signature scores for peri-bronchial and adventitial fibroblasts were more enriched in Subset 2, indicating important differences in fibroblast biology between subsets of IPF patients (Fig 5A). Gene signature scores for CTHRC1^+^ fibroblasts were not different between the two subsets. Taken together, this data indicated that the two subsets identified in GSE47460 (Kaminski-LGRC bulk expression cohort) [14–17], based on gene expression, also had concomitant differences in mesenchymal cell biology. Table 3 provides a short summary of cell type and representative gene expression changes associated with Subsets 1 and 2 in GSE47460 (Kaminski-LGRC bulk expression cohort) [14–17]. Based on the cell population changes described in Table 3, we will refer to Subset 1 as ‘Myeloid-enriched IPF’ and Subset 2 as ‘Ciliated epithelium-enriched IPF’.

**Table 3:** Summary of cellular and gene expression changes in patient subsets in GSE47460.

Subset in GSE47460	Associated cluster-specific cell type changes	Example gene expression changes
Subset 1.	Myeloid cell populations↑, mast cells↑, CTHRC1+ fibroblasts↑, pericytes↑, Alveolar fibroblasts subtypes↑	CCR2↑, CD11b↑, PPBP↑↑
Subset 2.	B cells/Plasma cells↑↑, Ciliated epithelium↑↑, Peribronchial fibroblasts↑, CTHRC1+ fibroblasts↑	MZB1↑, POU2AF1↑, FOXJ1↑↑

Column 2: ↑, moderately increased, ↑↑, strongly increased; Column 3: ↑, moderately upregulated over healthy (1.5-2x); ↑↑, strongly upregulated over healthy (>2x).

### Differential ligand-receptor networks as potential drivers of cell recruitment in IPF subsets

**Fig 6:**
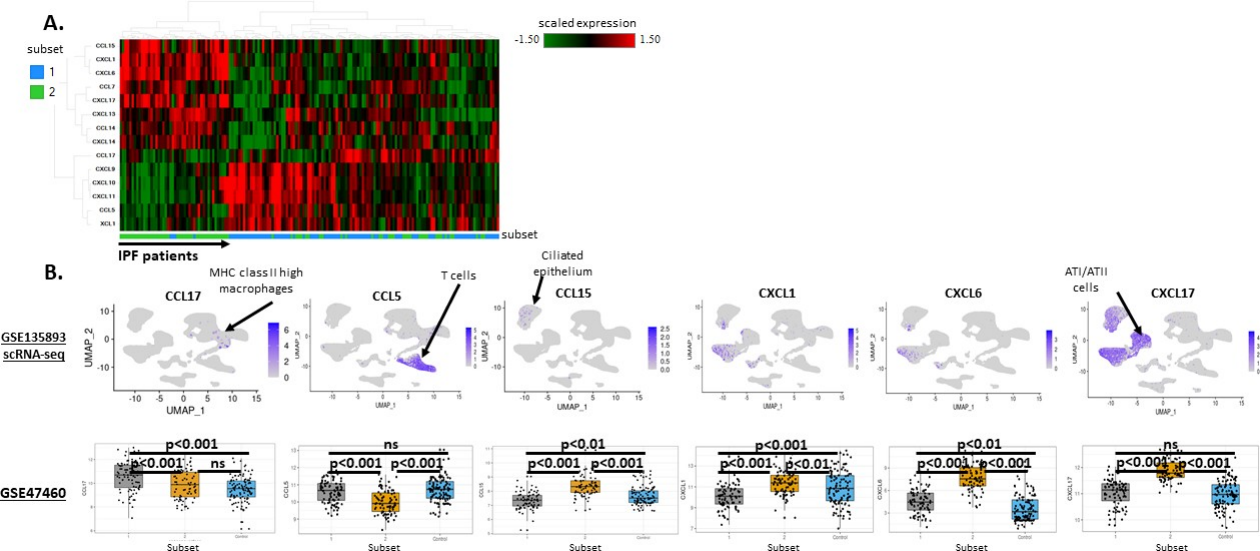
Evaluation of differential chemokine networks in IPF subsets. A. Hierarchical clustering of GSE47460 (Kaminski-LGRC bulk expression cohort) [14–17] IPF patients based on chemokines differentially expressed between IPF subsets. Absolute log FC >0.58 and adjusted p value<0.05 was used to define differentially expressed chemokines. x axis represents individual patients, y axis represents genes. Subsets are indicated in x axis color bar and legend of heatmap and correspond to classes shown in Fig 1A. B. Expression of chemokines detectable in GSE135893 (Kropski-Vanderbilt Univ single cell cohort) [24] scRNAseq data and in GSE47460 (Kaminski-LGRC bulk expression cohort) [14–17] IPF subsets. scRNAseq UMAP plots (top row) were generated using the ‘FeaturePlot’ function in R package Seurat. Each UMAP plot depicts the expression of the chemokine indicated. Color bars indicate scaled expression in each cell on the plot. Cell clusters correspond to clusters reported in S5 Fig. Bar plots (bottom row) depict the expression of the same chemokines in GSE47460 (Kaminski-LGRC bulk expression cohort) [14–17] IPF subsets. Adjusted p values are reported on plots.

We hypothesized that the differential cellular make-up in the Myeloid-enriched IPF subset as compared to the Ciliated epithelium-enriched IPF subset was likely due to the differential activation of chemokine and chemokine receptor networks. To test this hypothesis, first, we compared the expression patterns of chemokine ligands across the two subsets identified in GSE47460 (Kaminski-LGRC bulk expression cohort) [14–17]. As shown in Fig 6A, this analysis of GSE47460 (Kaminski-LGRC bulk expression cohort) [14–17] showed clustering of samples and chemokine ligands differentially expressed between the two IPF patient subsets. The Myeloid-enriched IPF subset (Subset 1) had increased expression of XCL1, CCL17, CCL5, CXCL9, CXCL10 and CXCL11, whereas the Ciliated epithelium-enriched IPF subset (Subset 2) had increased expression of CCL15, CXCL1, CXCL6, CCL7, CXCL17, CXCL13, CCL14 and CXCL14. We next examined the cellular origin of these chemokines using single cell data from Habermann et al. (GSE135893, Kropski-Vanderbilt Univ single cell cohort) [24]. Not all chemokines were detectable in scRNAseq data. Fig 6B shows detectable chemokine genes with differential expression between subsets of IPF patients. We found that CCL17 was primarily produced by MHC class II high macrophages and was higher in the Myeloid-enriched IPF subset. CCR4 is the receptor for CCL17 and is expressed by helper T cells, which may account for the increased numbers of T helper cells in the Myeloid-enriched IPF subset of patients (Fig 4B). CCL15 was primarily produced by ciliated epithelial cells, and was accordingly higher in the Ciliated epithelium-enriched IPF subset (Fig 6B).

**Fig 7:**
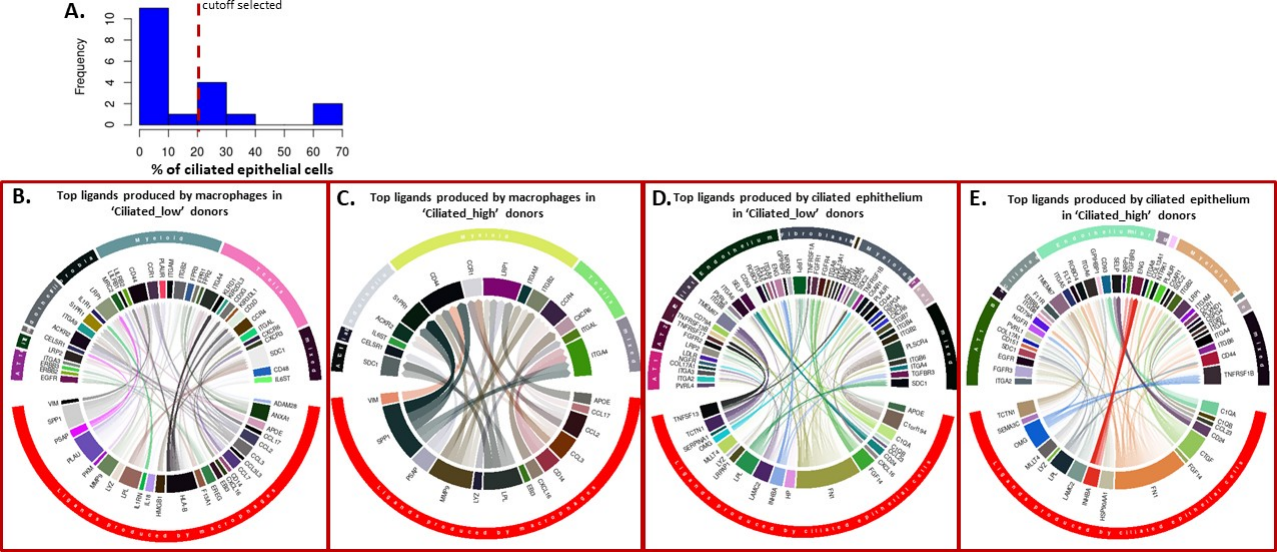
Ligand-receptor networks in ‘Ciliated_low’ and ‘Ciliated_high’ donors in GSE135893 (Kropski-Vanderbilt Univ single cell cohort) [24]. A. Histogram of distribution of percentage of ciliated epithelial cells in GSE135893 (Kropski-Vanderbilt Univ single cell cohort) [24] with the cutoff we selected indicated. B-E. Circular plots indicating ligand receptor interactions in subsets of patients in GSE135893 (Kropski-Vanderbilt Univ single cell cohort) [24]. On each circular plot, the top half of the circle represents cell types expressing the receptor for the ligand of the indicated cell type on the bottom half of the plot. Connections represent inferred active ligand-receptor pairs between types of cells. The thickness of the lines represents the relative level of expression of a given ligand/receptor. Transparency of the lines represents relative strength of the given ligand-receptor interaction as reported by the z score value calculated by PyMiner. B. Top ligands produced by macrophages (bottom half of circle) in ‘Ciliated_low’ patients and the top receptors they interact with (top half of circle with cell types expressing the receptor indicated). C. Top ligands produced by macrophages (bottom half of circle) in ‘Ciliated_high’ patients and the top receptors they interact with (top half of circle with cell types expressing the receptor indicated). D. Top ligands produced by ciliated epithelial cells (bottom half of circle) in ‘Ciliated_low’ patients and the top receptors they interact with (top half of circle with cell types expressing the receptor indicated). E. Top ligands produced by ciliated epithelial cells (bottom half of circle) in ‘Ciliated_high’ patients and the top receptors they interact with (top half of circle with cell types expressing the receptor indicated).

Additionally, we also conducted a transcriptome-wide analysis of single cell RNA seq data from Habermann et al. (GSE135893, Kropski-Vanderbilt Univ single cell cohort) [24] using the recently published ‘PyMiner’ approach for differential ligand-receptor expression [39]. This analysis confirmed expression of chemokine and chemokine receptor pairs using single cell data subsets and confirmed IPF subsetting into Myeloid-enriched and Ciliated epithelium-enriched IPF subsets and extended our analysis to additional ligand-receptor pairs active in subsets of IPF patients. The GSE135893 (Kropski-Vanderbilt Univ single cell cohort) [24] data set contained 19 IPF subjects and we separated these subjects into Myeloid-enriched and Ciliated epithelium-enriched IPF subsets using the percentage of all ciliated epithelial cells (i.e. sum of Ciliated_1, Ciliated_3, Diff_ciliated_15, Ciliated_28 populations in S8 Fig) from each subject (Fig 7A) to separate IPF subjects into ‘Ciliated_low’ (< 20% of cells are ciliated epithelial cells, analogous to the Myeloid-enriched IPF subset in GSE47460, Kaminski-LGRC bulk expression cohort) [14–17] and ‘Ciliated_high’ (> 20% of cells are ciliated epithelial cells, analogous to the Ciliated epithelium-enriched IPF subset in GSE47460, Kaminski-LGRC bulk expression cohort) [14–17] (S8 Fig) subsets. Our subsetting of the 19 donors in GSE135893 (Kropski-Vanderbilt Univ single cell cohort) [24] based on ‘Ciliated_low’ and ‘high’ criteria was validated based on significant differences between subsets in the percentages of myeloid populations, with the most significant differences between subsets in macrophages (S3 Table), thereby matching subset data in GSE47460 (Kaminski-LGRC bulk expression cohort) [14–17] (Fig 1). Pathway enrichment analysis based on differential gene expression between ‘Ciliated_high’ IPF patients and control samples or ‘Ciliated_low’ IPF patients and control samples in GSE135893 (Kropski-Vanderbilt Univ single cell cohort) [24] applied on this subsetting confirmed the activation of relevant pathways in the dataset (S2 Table). Additionally, we confirmed that percentages of relevant cell types followed the expected changes between subsets of IPF patients in GSE135893 (Kropski-Vanderbilt Univ single cell cohort, S8B Fig) [24]. Indeed, we detected significant differences in myeloid cells and a trend in endothelial cell percentages between subsets (with myeloid cells being enriched in the ‘Ciliated_low’ subset) similarly to the bulk RNA results shown above (S8 Fig). Full breakdown of individual donor-level percentages of all cell populations in GSE135893 (Kropski-Vanderbilt Univ single cell cohort) [24] between ‘Ciliated_high’ and ‘Ciliated_low’ IPF subsets is provided in S3 Table. We highlighted cell populations significantly different between ‘Ciliated_high’ and ‘Ciliated_low’ subsets in GSE135893 (Kropski-Vanderbilt Univ single cell cohort) [24] in S3 Table.

In our ligand-receptor analysis using PyMiner, we focused on differential ligands produced by cell populations that were significantly different in percentage between ‘Ciliated_low’ and ‘Ciliated_high’ subsets (specifically macrophage populations and ciliated epithelial cell populations, respectively). Fig 7B depicts circular diagrams with ligands produced by macrophages in ‘Ciliated_low’ subjects and Fig 7C depicts circular diagrams with ligands produced by macrophages in ‘Ciliated_high’ donors (Fig 7C). In Fig 7D, we depicted ligands produced by ciliated epithelium in ‘Ciliated_low’ donors, and in Fig 7E we depicted ligands produced by ciliated epithelium in ‘Ciliated_high’ donors (Fig 7E). We examined the top 10^th^ percentile of ligand-receptor pairs from macrophages and epithelial cells (based on z score output provided by PyMiner) in each subset and determined the expression of matching receptors from each condition for visualization purposes. As expected, significant differences in the profiles of inferred active ligand-receptor networks were detected. We confirmed these results using another ligand-receptor network approach, NicheNet [39] (S9 Fig). Overall, differential gene expression results derived from single cell and bulk RNA expression data suggested that a monocyte-macrophage chemoattractant axis (including potentially CCL2-CCR2 and CCL17-CCR4) was highly activated in ‘Ciliated_low’ (Myeloid-enriched IPF subset) patients and was possibly responsible for recruiting inflammatory macrophages in this subset, whereas ciliated epithelium-derived chemokine production (e.g. CCL15) may play an important role in cell recruitment in the Ciliated epithelium-enriched IPF subset of patients.

### Development of machine learning-based classifiers for distinguishing the Myeloid-enriched IPF subset versus the Ciliated epithelium-enriched subset

Because the two IPF patient subsets we identified in GSE47460 (Kaminski-LGRC bulk expression cohort) [14–17] may have a differential pathogenesis, our findings may have implications for treatment and disease progression. Therefore, we developed models to identify key features to distinguish the subsets. We used machine learning models with recursive feature elimination to 1.) identify the cell types that best distinguished the subsets using the gene expression signature scores described in Figs 3–5 and 2) identify gene expression that can be used to classify IPF patients into subsets. The two approaches offer distinct advantages for predicting subset membership: the first approach identifies biopsy histological features that may distinguish subsets, whereas the second approach permits development of RNA-based assays to distinguish subsets by measuring transcript levels from biopsy samples.

**Fig 8:**
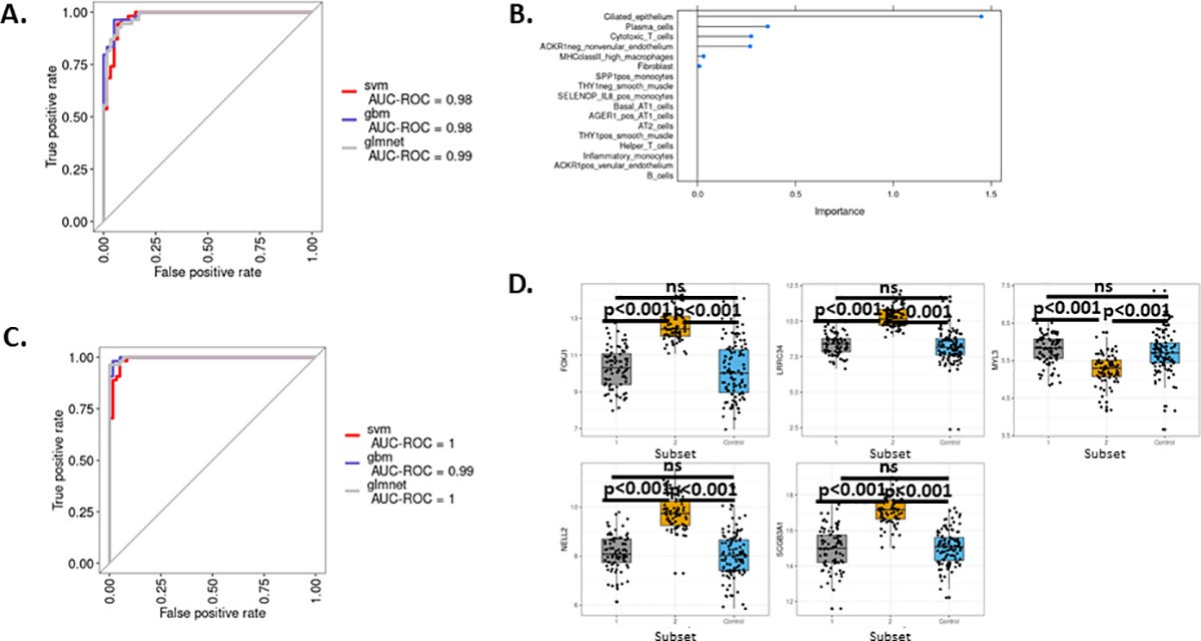
Building a machine learning-based classifier for distinguishing subclasses in GSE47460 (Kaminski-LGRC bulk expression cohort) [14–17]. A. ROC curve of classifier from three different methods used based on cell signature data. Legend indicates names of machine learning (svm, gbm, glmnet) used. B. Relative importance of cell types identified by the elastic net model sorted by importance. C. ROC curve of classifier from three different methods used based on gene expression data. Legend indicates names of machine learning (svm, gbm, glmnet) used. D. Expression values of top 5 genes identified by recursive feature elimination across subsets of patients in GSE47460 (Kaminski-LGRC bulk expression cohort) [14–17]. Adjusted p values are reported on plots.

We used support vector machines with a linear kernel, elastic net and a gradient boosting machine to create models for cell type-based classifiers and gene expression values. We trained our models on a randomly selected 70% of IPF patients in GSE47460 (Kaminski-LGRC bulk expression cohort) [14–17] and used the remaining 30% as a validation set with 5-fold cross-validation. All three methods (linear kernel, elastic net and gradient boosting) produced high accuracy models with AUROC values > 0.95 (Fig 8A for cell signature scores and 8C for gene expression values). The cell signature approach identified ciliated epithelium, plasma cells, cytotoxic T cells and ACKR1 negative endothelium as the most important features separating the two subsets of IPF (Fig 8B). Recursive feature elimination using gene expression values identified FOXJ1, NELL2, SCGB3A1, LRRC34 and MYL3 as the top five most predictive genes (Fig 8D).

### Differences in pirfenidone response signature between subsets of IPF patients

**Fig 9:**
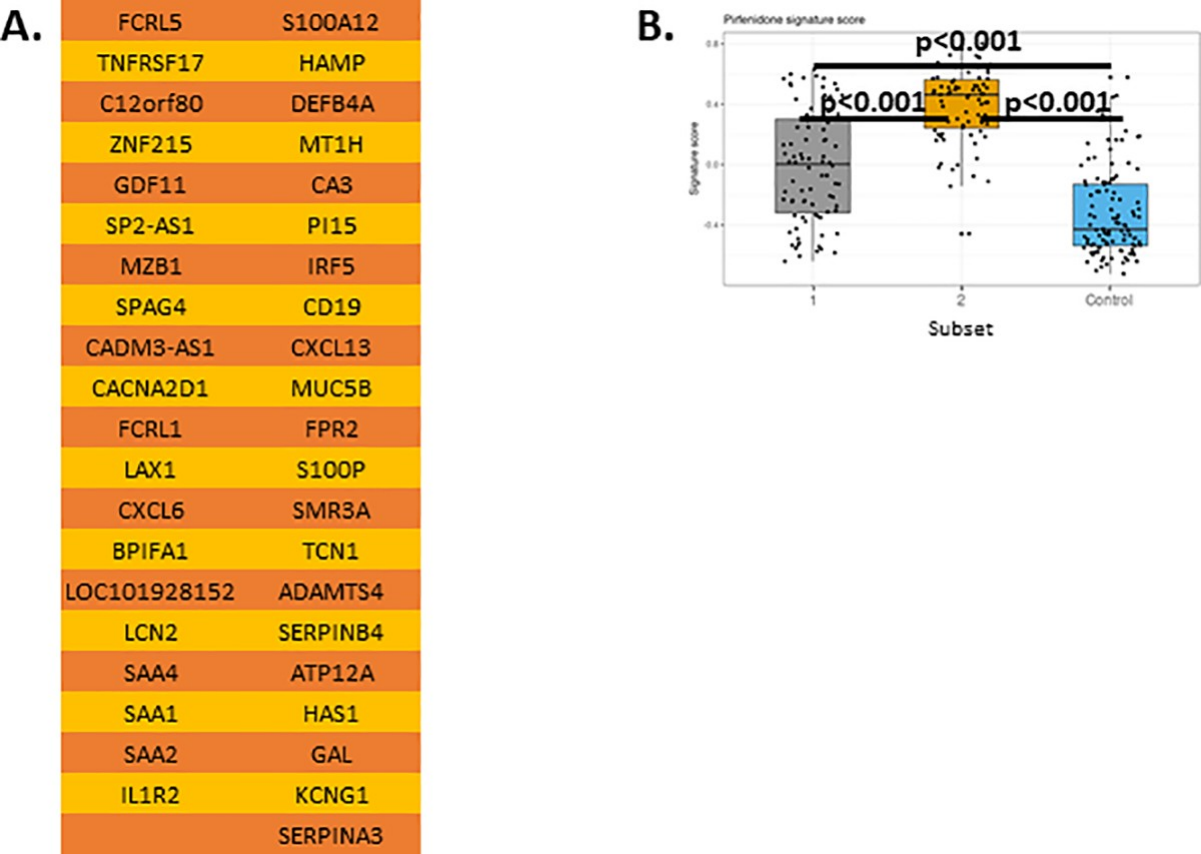
Expression of a pirfenidone response gene signature differs between IPF subsets. A. Pirfenidone response signature from reference [42]. B. Gene signature scores in GSE47460 (Kaminski-LGRC bulk expression cohort) [14–17]. Adjusted p values are reported on plots.

Finally, we asked whether the two subsets of IPF patients would respond differentially to approved IPF therapies. Currently, there are two FDA-approved therapies available for IPF patients, pirfenidone and nintedanib [3, 4]. We developed a lung pirfenidone signature using genes *downregulated* in response to pirfenidone in lung homogenates [42] (Fig 9A). Applying this gene signature to the two subsets we identified in GSE47460 (Kaminski-LGRC bulk expression cohort) [14–17], we found that pirfenidone-responsive genes were upregulated in both subsets of IPF patients; however, pirfenidone-responsive genes were more significantly upregulated in the Ciliated epithelium-enriched IPF subset (Fig 9B). These results suggested that the Ciliated epithelium-enriched IPF subset may be more responsive to pirfenidone as compared to the Myeloid-enriched IPF subset.

## Discussion

We used a data-driven, unsupervised clustering of RNA expression data from IPF patient lung samples, that was reproducible across patient cohorts and was associated with changes in the cellular composition of the lungs in IPF. We believe this study provides novel ideas on differential mechanisms of pathogenesis in this heterogeneous disease. We used single cell RNA sequencing data to uncover subpopulations of both mesenchymal and hematopoietic cell populations associated with disease pathogenesis [19, 6, 45–47]. The throughput of scRNAseq studies limits sample sizes; therefore, we devised an analysis pipeline to bridge this gap by applying single cell RNA-based signature analysis to gene expression data derived from whole tissue gene expression. Through this analysis, we identified key alterations in cellular compositions and molecular mechanisms specific for 2 subsets of IPF patients. Furthermore, we identified potential biomarkers through the use of well-established machine learning techniques to develop classifiers based on both cell type signatures and gene expression values. Overall, we believe this body of work will help with the development of IPF diagnostics and deepen our understanding of cell types involved in the pathogenesis of IPF in different subsets of patients.

Prior studies suggested that both plasma cells and T cells are associated with disease progression [11–13], and B cells and plasma cells are enriched in IPF lung tissue and plasma cell gene expression is associated with faster disease progression and poorer survival [13]. These findings were confirmed using additional patient cohorts and different methodologies [48]. However, the nature of plasma cell and IPF-specific autoantibody involvement in IPF is unclear and current studies do not provide a mechanism to connect plasma cell and autoantibody increases to IPF pathogenesis [49, 50]. The subset associated with the strongest B cell/plasma cell signature was the same subset (Ciliated epithelium-enriched IPF subset; Subset 2) associated with a *decrease* in cytotoxic T cells and helper T cells. Similar to increases in B cells/plasma cells, decreases in T cell responses have been shown to be associated with a poor prognosis in IPF [11, 51]. Evaluation of the expression of genes suggested to be prognostic in IPF [43] suggested that the Ciliated epithelium-enriched IPF subset represented the subset of patients with more severe disease. Gene expression across the two subsets did not suggest an association with acute exacerbations of IPF; for example, some published markers of acute IPF exacerbations (MMP1, MMP7) were higher in Subset 2, while others (AGER, DEFA3) were lower in Subset 2 or not significantly different (COL1A2, CCNA2) [52]. Additionally, we found that 3 out of the 4 markers (*GREM1*, *MMP7*, *CTHRC1* and *FHL2*) identified by Kaminski and colleagues [43] as having a significant negative correlation with %DlCO and as markers that separate IPF patients by disease severity and predicted progression were expressed at higher levels in ‘Ciliated epithelium-enriched’ patients of GSE47460 (Kaminski-LGRC bulk expression cohort) [14–17] (S2 Fig), potentially indicating a differential prognosis for the two subsets of IPF patients. Clinical follow up data will be valuable to determine whether the Ciliated epithelium-enriched IPF subset is associated with a worse prognosis and more likely to have acute IPF exacerbations.

A potential concern related to our study is that the subsets detected in our analysis were associated with differential sampling of lung tissue in each study. There are several lines of evidence disputing this conclusion including the fact that the pattern of IPF patient subsets we detected were observed across multiple independent patient cohorts and using different technologies (bulk RNA sequencing and scRNAseq, references [10, 12]). Additionally, we re-analyzed samples from GSE124685 (Kaminski-Yale Univ bulk progression RNA cohort) [12], a study that analyzed various stages of IPF lungs by bulk RNA-sequencing. This study analyzed a small (n = 10) number of IPF donors and sampled their lungs in various anatomical locations to obtain transcriptional profiles of IPF lungs in various stages of fibrosis. Key markers identified in this study such as CCR2, ITGAM, FOXJ1 and SNTN did show significant changes by location of the lung samples [12]. It is also possible that the differences between subsets were due to differences in the stage of disease when tissue was sampled. Although we currently have no way of unequivocally determining whether the subsets were at different stages of disease (i.e. Subset 1 progresses into Subset 2), we think this is unlikely because the Myeloid-enriched IPF subset had a higher overall fibroblast gene expression signature compared to the Ciliated epithelium-enriched IPF subset (Fig 3). CTHRC1^+^ fibroblasts were recently shown to be present in fibrotic lungs [19] and were suggested to be pathogenic based on the high expression of several well-known pro-fibrotic mediators and extracellular matrix components. However, CTHRC1^+^ fibroblasts were not differentially expressed in the two subsets (Figs 3–5). Besides CTHRC1^+^ fibroblasts, several well-known profibrotic genes were similarly expressed between the two subsets (Fig 2). Additionally, Subset 1 patients expressed lower levels of genes shown to be prognostic of disease progression [43], and the Myeloid-enriched IPF subset was associated with increased mast cells compared to the Ciliated epithelium-enriched IPF subset (S6 Fig, ‘MC_27’). Mast cells have been shown to be associated with a subset of IPF patients with a milder prognosis [53], further suggesting that the subsets we identified may have a different pathogenesis and prognosis. Taken together, these results suggested that Subsets 1 and 2 are not different stages of IPF but rather are subsets of IPF with different underlying pathologies and disease severity. This question can only be definitively answered using longitudinal gene expression data.

The Myeloid-enriched IPF subset was characterized by the presence of increased myeloid cell gene expression. Myeloid cells have been shown to be significant contributors to the development of fibrosis [54–56], and increases in SPP1-producing monocytes and macrophages were shown to be a hallmark of IPF pathogenesis [47]. Our analysis indicated that SPP1-producing monocytes along with other subsets of CD14^+^ cells were increased as compared to control samples in the Myeloid-enriched IPF subset but not in the Ciliated epithelium-enriched IPF subset (Fig 4C). This difference in macrophage numbers was associated with a significant, albeit small, increase in CCL2-expressing alveolar fibroblasts in the Myeloid-enriched IPF subset (Fig 5B) and increased expression of CCR2, the receptor for CCL2. We analyzed receptor-ligand interactions in IPF single cell data between myeloid cells and fibroblasts and found that myeloid cells potentially provide important ligands for the activation of fibroblasts and vice versa (Figs 6 and 7, see below). With the accumulation of more scRNAseq data, an essential question to ask will be if different ligand-receptor interactions contribute to the pathogenesis of IPF in the Myeloid-enriched IPF subset.

We extended the receptor-ligand analysis to better understand all potential ligand-receptor changes between subsets of patients; this confirmed the differential activation of the CCL2-CCR2 ligand-receptor pair in the Myeloid-enriched IPF subset and revealed additional major changes in active receptor-ligand interactions between Subsets 1 and 2. Notable examples included the predicted activation of the EREG-EGFR ligand-receptor in the Myeloid-enriched IPF subset and the predicted activation of CTGF signaling and the high level expression of CXCL13 in the Ciliated epithelium-enriched IPF subset (Fig 7B–7E). EGFR overexpression has been shown to be a hallmark of IPF [57] and EGFR activation has been shown to contribute to fibrosis in the bleomycin model of IPF [58]. As such, EGFR inhibition may represent an attractive therapeutic strategy for the Myeloid-enriched IPF subpopulation of IPF patients. Other studies have shown that CTGF is a key contributor to fibroblast activation and IPF [59]. The CTGF-blocking antibody pamrevlumab was beneficial in a recently completed phase II clinical trial in IPF [60]. A key question that our results may address is whether the Ciliated epithelium-enriched IPF subset responds better to pamrevlumab treatment. CXCL13 is a major chemokine responsible for the recruitment of antibody-producing cells and formation of germinal centers [61]. B cells and plasma cells are enriched in IPF lung tissue, plasma cell gene expression is associated with faster disease progression and poorer survival [13], and some IPF patients are responsive to B cell depletion with rituximab [62]. Therefore, it would be of interest to see whether rituximab responsiveness is associated with the subset of patients with high levels of B cells/plasma cells in the Ciliated epithelium-enriched IPF subset.

One of the major differences between Myeloid-enriched and Ciliated epithelium-enriched subsets of IPF patients was the expression of genes associated with ciliated epithelium as well as the increased expression of MUC5B in Subset 2. One study has suggested that ciliated epithelium is an important driver of IPF pathogenesis [63]. Additionally, MUC5B is a reproducible susceptibility locus identified in IPF genome-wide association studies (GWAS) [5–7]. Polymorphisms in the MUC5B promoter were shown to be associated with different levels of MUC5B expression [64–66]. Our analysis indicated that MUC5B upregulation was not a uniform feature of all IPF patients and was associated with ciliated epithelium abnormalities. An interesting question that arises from this analysis is whether patients in the Ciliated epithelium-enriched IPF subset are enriched in MUC5B polymorphisms associated with the pronounced upregulation of MUC5B mRNA. The potential connection between MUC5B upregulation and increased CTGF production found in our study is largely unexplored in the literature. Differential MUC5B production may also be valuable as a biomarker since MUC5B is routinely measured from sputum [67, 68]. Exploring the feasibility and value of MUC5B as a biomarker for differentiating the two subsets of IPF patients is worth considering.

We also developed biomarkers to distinguish IPF patient subsets based on either cellular alterations or changes in gene expression. Through this work, we generated a list of potential biomarkers to separate IPF subsets with high accuracy (Fig 8). Using the methods described herein, we found that several of the cell populations different across subsets may also be used as accurate predictors of IPF patient subset (Fig 8). Additionally, we were also able to find a gene set that may function as a predictor of IPF patient subset (Fig 8D). After further validation of our results, it will be essential to develop markers that reliably identify what subset individual patients belong to, i.e. to stratify them into the Myeloid-enriched (Subset 1) or Ciliated epithelium-enriched (Subset 2) subsets. Some of the top 5 genes in our classifier (Fig 8D; FOXJ1, LRRC34, MYL3, NELL2, SCGB3A1) have known relevance to the biology of ciliated epithelium (e.g. FOXJ1 is a key transcription factor in the development of cilia [69] and LRRC34 is a candidate causative gene in Mendelian disorders of cilium development [70]).

Additionally, we presented a patient stratification hypothesis for one of the currently FDA-approved treatments for IPF, pirfenidone. We showed that our Ciliated epithelium-enriched subset presented with significantly higher pirfenidone-responsive gene expression (Fig 9). These findings may lead to new hypotheses about differential patient treatment of IPF with pirfenidone and suggest a similar approach to the development of biomarkers for other approved therapies for IPF, such as nintedanib.

Our study has several strengths, including connecting alterations in cellular composition to gene expression and offering hypotheses on the differential pathogenesis underlying subsets of patients in IPF; however, it also has several limitations. First, although we used the best available method to assess consensus clustering performance (Proportion of Ambiguous Clustering (PAC) score, [26]), determining the optimal number of clusters from consensus clustering methods has known limitations [26]. It is possible that there is hidden sub-structure in the clusters detected and with a larger number of samples additional subsets could be discovered. Second, the number of donors in single cell RNA sequencing studies used to generate gene expression reference matrices for deconvolution of bulk data are small. Third, there are cell populations not reflected in the single cell RNA sequencing data (such as neutrophil granulocytes) that cannot be estimated in the bulk gene expression data.

Also, we used scores determined by gene set enrichment to estimate levels of cell type enrichment; as such, the scores calculated represent cell type-specific signatures and are not a direct measurement of each cell type. Despite this potential limitation, we believe that we used the most relevant GSVA method to determine cell type-specific gene signature scores; GSVA offers distinct advantages over calculating gene signature scores due to its efficient ranking and outlier smoothing algorithms [33, 34].

In addition, we believe that the conclusions we made using gene signatures developed from the scRNAseq datasets are also supported by the observation that the correlation between GSVA-signature scores calculated from total lung suspension datasets GSE132771 (Sheppard-UCSF single cell cohort) and GSE136893 (Kropski-Vanderbilt Univ single cell cohort) are low across the dataset (S3C and S5B Figs). Although we observed higher correlation values using the ‘Lineage sorted’ dataset from GSE132771 (Sheppard-UCSF single cell cohort), we believe this does not change the main conclusions derived from the results. For example, in Fig 5B there was a high correlation and overlap between THY1high_alv_fib_0 (Alveolar_0) and THY1neg_alv_fib_5 (Alveolar_5) fibroblasts and gene signature scores for these populations (along with the other two alveolar fibroblast populations) across subsets. Therefore, using this example, we found that Subset 1 (Myeloid cell-enriched subset) showed an enrichment in gene signature scores for all alveolar fibroblast populations (Fig 5B) as compared to Control/Subset 2 (Ciliated epithelium-enriched subset) but a decrease in adventitial and peribronchial fibroblasts (Fig 5A).

Another limitation of our study is that it represents ‘hypothesis generation’ and lacks experimental validation. Unfortunately, we were unable to link and validate our findings to histopathological and longitudinal clinical and gene expression data. Future datasets may answer the question of whether the changes we observed based on gene expression are reflected in cellular changes observable by other methods and if gene expression differences are relevant to prediction of clinical disease course in IPF. However, we believe that our hypotheses have generated valuable insights despite this shortcoming as our results provide testable ideas with suggested associated biomarkers.

## Conclusions

In conclusion, we developed an analysis pipeline to subset IPF patients in a data-driven, unsupervised manner and demonstrated an association of cellular changes with gene expression in the two identified subsets. We believe this work provides novel insights into the pathogenesis of IPF and provides testable hypothesesabout differential alterations of cellular composition of the lung in subsets of IPF patients in this difficult-to-treat disease.

## Supporting information

S1 Fig A. PAC scores as a function of number of clusters (k) calculated based on consensus clustering results in GSE47460 (Kaminski-LGRC bulk expression cohort) [14–17]. B. PAC scores as a function of number of clusters (k) calculated based on consensus clustering results in GSE134692 (BMS bulk RNA-seq cohort) [18]. C. Distribution of patient subsets from Fig 1A across IPF samples overlapping or non-overlapping between GSE47460 (Kaminski-LGRC bulk expression cohort) [14–17] and GSE32537 (Schwartz-Univ of Colorado bulk expression cohort) [10]. D. PAC scores as a function of number of clusters (k) calculated based on consensus clustering results using the 75 unique samples (not overlapping with GSE32537) from GSE47460 (Kaminski-LGRC bulk expression cohort) [14–17].
(TIF)

S2 Fig Expression of genes identified in [43] to be associated with disease progression in subsets of GSE47460 (Kaminski-LGRC bulk expression cohort) [14–17]. Adjusted p values are reported on plots.
(TIF)

S3 Fig Cell types in GSE132771 (Sheppard-UCSF single cell cohort) [19]. Clustering was performed using R package Seurat and cell types were identified using known markers. A. Total lung cell suspension. SPP1_monocytes_0: SPP1^+^ monocytes; Infl_monocytes_1: Inflammatory monocytes; ACKR1pos_endo_2: ACKR1^+^ endothelial cells; ACKR1neg_endo_3: ACKR1- endothelial cells; Fibroblasts_4: Fibroblasts; AT2_5 and AT2_23: Alveolar epithelial cell type II subpopulations; Th_6: helper T cells; Pericytes_7 and Pericytes_22: Pericyte subpopulations; HLAhigh_mac_8 and HLAhigh_mac_10: HLA class II high macrophage subpopulations; Sm_9: smooth muscle cells; Bcells_11 and Bcells_21: B cell subpopulations; Tc_12: cytotoxic T cells; AT1_13: Alveolar epithelial cell type I; PC_14: Plasma cells; Endo_15 and Endo_24: endothelial cell subpopulations; Ciliated_16: ciliated epithelial cells; Monocytes_17 and Monocytes_18: Monocyte subpopulations. B. Lineage sorted cells. THY1high_alv_fib_0: THY1 high alveolar fibroblasts; THY1pos_sm_1: THY1^+^ smooth muscle; THY1neg_sm_2: THY1- smooth muscle; CTHRC1pos_3: CTHRC1^+^ fibroblasts; Adventitial_4: Adventitial fibroblasts; THY1neg_alv_fib_5: THY1- alveolar fibroblasts; Pericytes_6: Pericytes; Peribronchial_7: Peribronchial fibroblasts; Sm_8 and Sm_13: smooth muscle cell subpopulations; Alveolar_9 and Alveolar_10: Alveolar fibroblast subpopulations; Epi_11: Epithelial cells; Hematopoietic_12 and Hematopoietic_14: Hematopoietic cells. C. Heatmap (left panel) and correlation matrix (right panel) in GSE47460 of genes included in the signature derived from the ‘Total lung cell suspension’ (shown in panel A) dataset across each cluster shown in panel A. D. Heatmap (left panel) and correlation matrix (right panel) in GSE47460 of genes included in the signature derived from the ‘Lineage sorted’ (shown in panel B) dataset across each cluster shown in panel B.
(ZIP)

S4 Fig A. Expression of various B cell, plasma cell and myeloid markers in GSE47460 (Kaminski-LGRC bulk expression cohort) [14–17] subsets. B. Expression of ciliated epithelium cell markers in GSE47460 (Kaminski-LGRC bulk expression cohort) [14–17] subsets. Adjusted p values are reported on plots.
(TIF)

S5 Fig A. Cell type labels used based on re-analysis of IPF and healthy control data from GSE135893 (Kropski-Vanderbilt Univ single cell cohort) [24]. Clustering was performed using R package Seurat and cell types were identified using known markers. Ciliated_0 and Ciliated_1: Ciliated epithelial cell subpopulations; AT2_2, AT2_13, AT2_29, AT2_30: Alveolar epithelial cell type II subpopulations; SPP1_mac_3: SPP1^+^ monocytes/macrophages; C1QA_mac_4, C1QA_mac_5, C1QA_mac_9, C1QA_mac_12: C1QA^+^ macrophage subpopulations; Mono_7, Mono_21: Monocyte subpopulations; Tc_8: cytotoxic T cells; Th_10: helper T cells; AT1_11, MUC5Bpos_AT1_15, Basal_AT1_17: Alveolar epithelial cell type I subpopulations; ACKR1_pos_endo_14: ACKR1^+^ endothelial cells; ACKR1_neg_endo_16 and ACKR1_neg_endo_20: ACKR1- endothelial cell subpopulations; Diff_cil_18: Differentiating ciliated epithelial cells; Fibroblasts_19 and Fibroblasts_23: Fibroblast subpopulations; Sm_26: smooth muscle; Prolif_mac_22: Proliferating macrophages; Ly_endo_24: Lymphatic endothelium; Bcells_25: B cells; PC_28: Plasma cells; MC_27: mast cells; Mesothelial_31: mesothelial cells. B. Heatmap (left panel) and correlation matrix (right panel) in GSE47460 (Kaminski-LGRC bulk expression cohort) of genes included in the signature derived from the dataset shown in panel A.
(ZIP)

S6 Fig Cell signature scores in GSE47460 (Kaminski-LGRC bulk expression cohort) [14–17] using cell type signatures based on GSE135893 (Kropski-Vanderbilt Univ single cell cohort) [24]. Only cell types with relevance to subsetting are shown. Nomenclature of cell types follows S5 Fig.
(TIF)

S7 Fig Cell signature scores in GSE134692 (BMS bulk RNA-seq cohort) [18] using cell type signatures based on GSE132771 (Sheppard-UCSF single cell cohort) [19]. Only cell types with relevance to subsetting shown. Nomenclature of cell types follows S3 Fig. **A.** Non-hematopoietic populations from S3A Fig. **B.** Hematopoietic populations from S3A Fig. **C.** Cell populations from S3B Fig.
(ZIP)

S8 Fig A. IPF samples in GSE135893 (Kropski-Vanderbilt Univ single cell cohort) [24] divided by the % of total ciliated cells in the data as shown in Fig 7A. SPP1pos_macs_0: SPP1^+^ monocytes/macrophages; Ciliated_1, Ciliated_3 and Ciliated_28: Ciliated epithelial cell subpopulations; C1QA_mac_2 and C1QA_mac_6: C1QA positive macrophage subpopulations; AT1_4, AT1_9, AT1_11, AT1_26: Alveolar epithelial cell type I subpopulations; AT2_5 and AT2_24: Alveolar epithelial cell type II subpopulations; ACKR1pos_endo_7: ACKR1^+^ endothelial cells; Monocytes_8: monocytes; Th10: helper T cells; Macs_12, Macs_22 and Macs_27: Macrophage subpopulations; Tc_13: cytotoxic T cells; HAS1_fibro_14: HAS1 positive fibroblasts; Diff_ciliated_15: differentiating ciliated epithelial cells; ACKR1neg_endo_16: ACKR1- endothelial cells; Fibroblasts_17 and Fibroblasts_29: Fibroblast subpopulations; Prolif_macs_18: Proliferating macrophages; Ly_endo_19: Lymphatic endothelium; Sm_20: smooth muscle; Bcells_21: B cells; PC_23: Plasma cells; MC_25: Mast cells. B. Differences in the percentage of Ciliated cells, Total myeloid cells and Endothelial cells between ‘Ciliated_low’ and ‘Ciliated_high’ subsets in GSE135893 (Kropski-Vanderbilt Univ single cell cohort) [24]. Percentages were calculated using cell numbers of the cell type indicated divided by the total number of cells in the data (subset based on Ciliated epithelial cells). Adjusted p values are reported on plots.
(TIF)

S9 Fig Top differentially active ligand-receptor network as predicted by NicheNet between ‘Ciliated_low’ and ‘Ciliated_high’ donors in GSE135893 (Kropski-Vanderbilt Univ single cell cohort) [24]. Size of circle indicated percent of cells gene on x axis is expressed in; color represents relative expression level. Nomenclature of cell clusters follows S8 Fig.
(TIF)

S1 Table (XLSX)

S2 Table (XLSX)

S3 Table (XLSX)
