# Supplementary figures and images for: Lung gene expression and single cell analyses reveal two subsets of idiopathic pulmonary fibrosis (IPF) patients associated with different pathogenic mechanisms
Source: PLoS One. 2021 Mar 23;16(3):e0248889. doi: 10.1371/journal.pone.0248889 (PMC7987152; doi:10.1371/journal.pone.0248889)

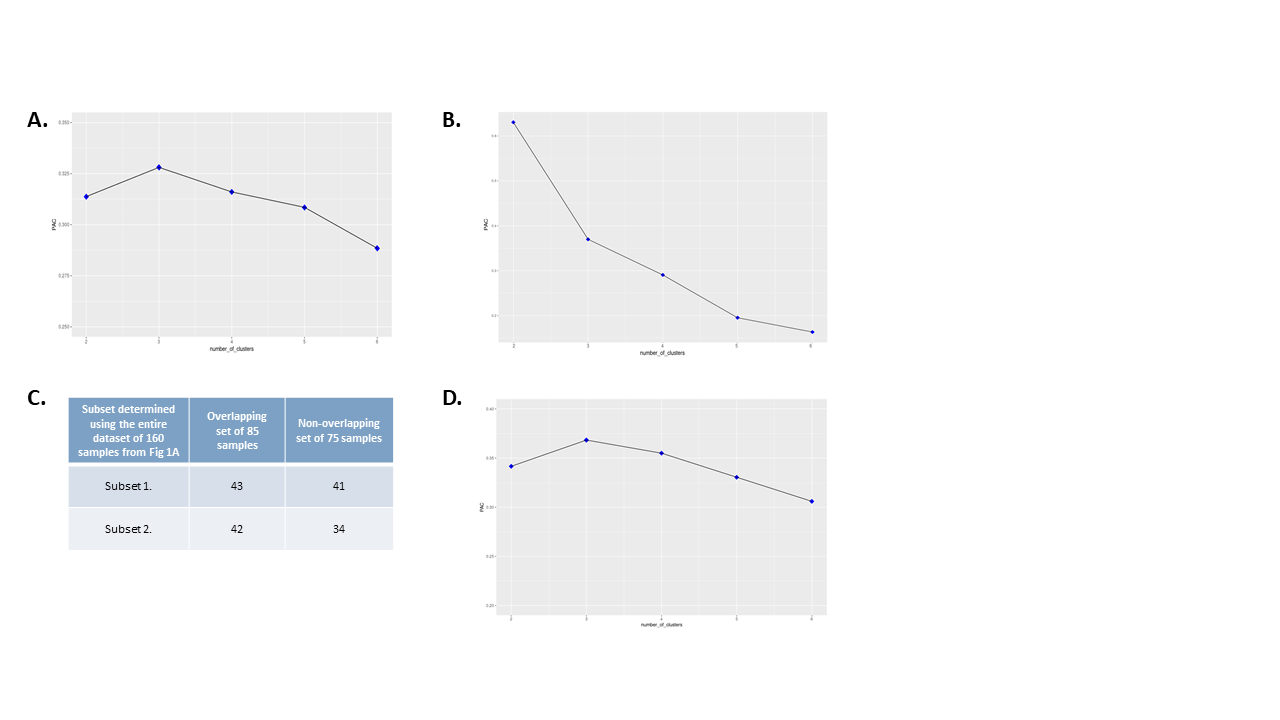

Supplement: S1 Fig — A. PAC scores as a function of number of clusters (k) calculated based on consensus clustering results in GSE47460 (Kaminski-LGRC bulk expression cohort) [14–17]. B. PAC scores as a function of number of clusters (k) calculated based on consensus clustering results in GSE134692 (BMS bulk RNA-seq cohort) [18]. C. Distribution of patient subsets from Fig 1A across IPF samples overlapping or non-overlapping between GSE47460 (Kaminski-LGRC bulk expression cohort) [14–17] and GSE32537 (Schwartz-Univ of Colorado bulk expression cohort) [10]. D. PAC scores as a function of number of clusters (k) calculated based on consensus clustering results using the 75 unique samples (not overlapping with GSE32537) from GSE47460 (Kaminski-LGRC bulk expression cohort) [14–17]. (TIF) [file pone.0248889.s001.tif]

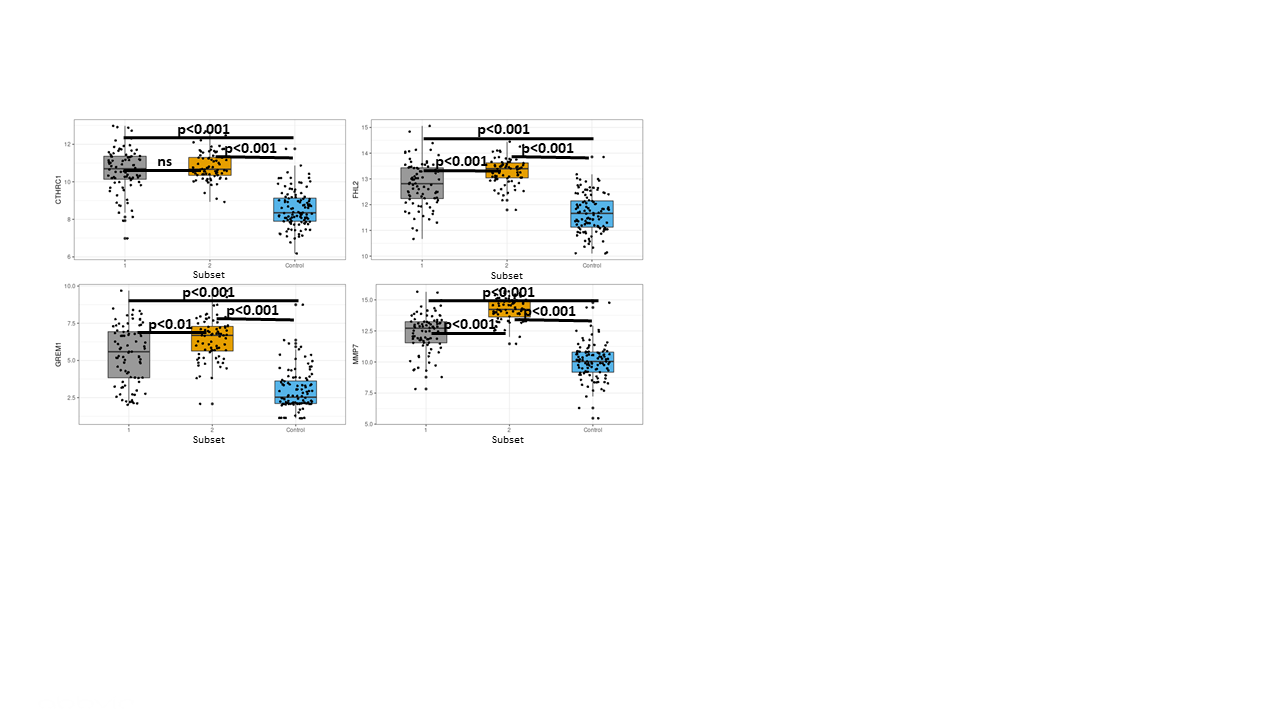

Supplement: S2 Fig — Adjusted p values are reported on plots. (TIF) [file pone.0248889.s002.tif]

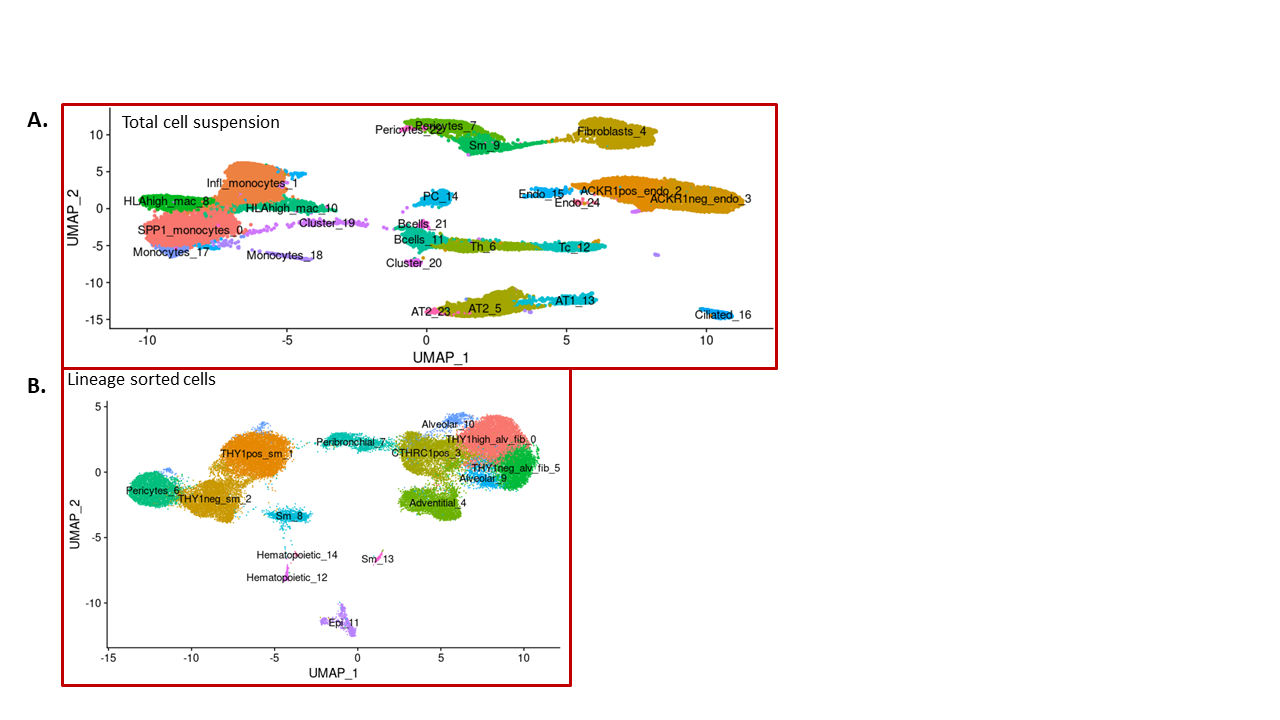

Supplement: S3 Fig — Clustering was performed using R package Seurat and cell types were identified using known markers. A. Total lung cell suspension. SPP1_monocytes_0: SPP1+ monocytes; Infl_monocytes_1: Inflammatory monocytes; ACKR1pos_endo_2: ACKR1+ endothelial cells; ACKR1neg_endo_3: ACKR1- endothelial cells; Fibroblasts_4: Fibroblasts; AT2_5 and AT2_23: Alveolar epithelial cell type II subpopulations; Th_6: helper T cells; Pericytes_7 and Pericytes_22: Pericyte subpopulations; HLAhigh_mac_8 and HLAhigh_mac_10: HLA class II high macrophage subpopulations; Sm_9: smooth muscle cells; Bcells_11 and Bcells_21: B cell subpopulations; Tc_12: cytotoxic T cells; AT1_13: Alveolar epithelial cell type I; PC_14: Plasma cells; Endo_15 and Endo_24: endothelial cell subpopulations; Ciliated_16: ciliated epithelial cells; Monocytes_17 and Monocytes_18: Monocyte subpopulations. B. Lineage sorted cells. THY1high_alv_fib_0: THY1 high alveolar fibroblasts; THY1pos_sm_1: THY1+ smooth muscle; THY1neg_sm_2: THY1- smooth muscle; CTHRC1pos_3: CTHRC1+ fibroblasts; Adventitial_4: Adventitial fibroblasts; THY1neg_alv_fib_5: THY1- alveolar fibroblasts; Pericytes_6: Pericytes; Peribronchial_7: Peribronchial fibroblasts; Sm_8 and Sm_13: smooth muscle cell subpopulations; Alveolar_9 and Alveolar_10: Alveolar fibroblast subpopulations; Epi_11: Epithelial cells; Hematopoietic_12 and Hematopoietic_14: Hematopoietic cells. C. Heatmap (left panel) and correlation matrix (right panel) in GSE47460 of genes included in the signature derived from the ‘Total lung cell suspension’ (shown in panel A) dataset across each cluster shown in panel A. D. Heatmap (left panel) and correlation matrix (right panel) in GSE47460 of genes included in the signature derived from the ‘Lineage sorted’ (shown in panel B) dataset across each cluster shown in panel B. (ZIP) [file pone.0248889.s003.zip › S3A-B_Fig.tif]

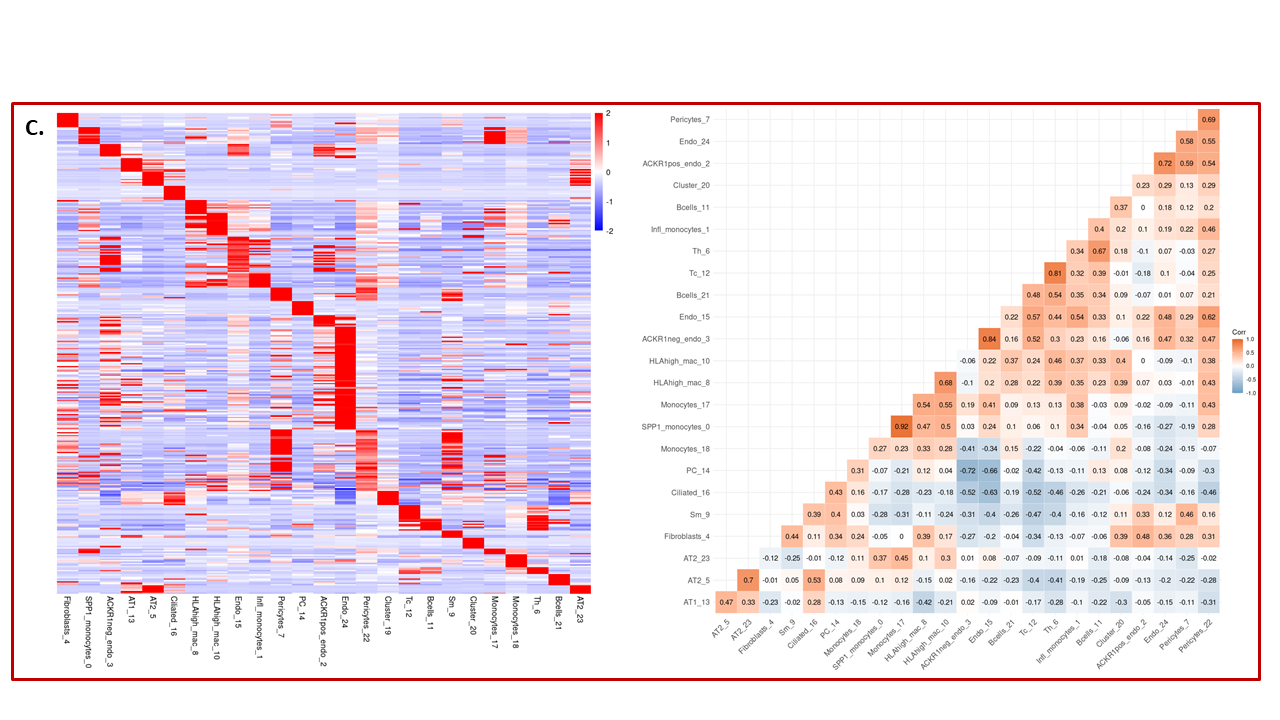

Supplement: S3 Fig — Clustering was performed using R package Seurat and cell types were identified using known markers. A. Total lung cell suspension. SPP1_monocytes_0: SPP1+ monocytes; Infl_monocytes_1: Inflammatory monocytes; ACKR1pos_endo_2: ACKR1+ endothelial cells; ACKR1neg_endo_3: ACKR1- endothelial cells; Fibroblasts_4: Fibroblasts; AT2_5 and AT2_23: Alveolar epithelial cell type II subpopulations; Th_6: helper T cells; Pericytes_7 and Pericytes_22: Pericyte subpopulations; HLAhigh_mac_8 and HLAhigh_mac_10: HLA class II high macrophage subpopulations; Sm_9: smooth muscle cells; Bcells_11 and Bcells_21: B cell subpopulations; Tc_12: cytotoxic T cells; AT1_13: Alveolar epithelial cell type I; PC_14: Plasma cells; Endo_15 and Endo_24: endothelial cell subpopulations; Ciliated_16: ciliated epithelial cells; Monocytes_17 and Monocytes_18: Monocyte subpopulations. B. Lineage sorted cells. THY1high_alv_fib_0: THY1 high alveolar fibroblasts; THY1pos_sm_1: THY1+ smooth muscle; THY1neg_sm_2: THY1- smooth muscle; CTHRC1pos_3: CTHRC1+ fibroblasts; Adventitial_4: Adventitial fibroblasts; THY1neg_alv_fib_5: THY1- alveolar fibroblasts; Pericytes_6: Pericytes; Peribronchial_7: Peribronchial fibroblasts; Sm_8 and Sm_13: smooth muscle cell subpopulations; Alveolar_9 and Alveolar_10: Alveolar fibroblast subpopulations; Epi_11: Epithelial cells; Hematopoietic_12 and Hematopoietic_14: Hematopoietic cells. C. Heatmap (left panel) and correlation matrix (right panel) in GSE47460 of genes included in the signature derived from the ‘Total lung cell suspension’ (shown in panel A) dataset across each cluster shown in panel A. D. Heatmap (left panel) and correlation matrix (right panel) in GSE47460 of genes included in the signature derived from the ‘Lineage sorted’ (shown in panel B) dataset across each cluster shown in panel B. (ZIP) [file pone.0248889.s003.zip › S3C_Fig.tif]

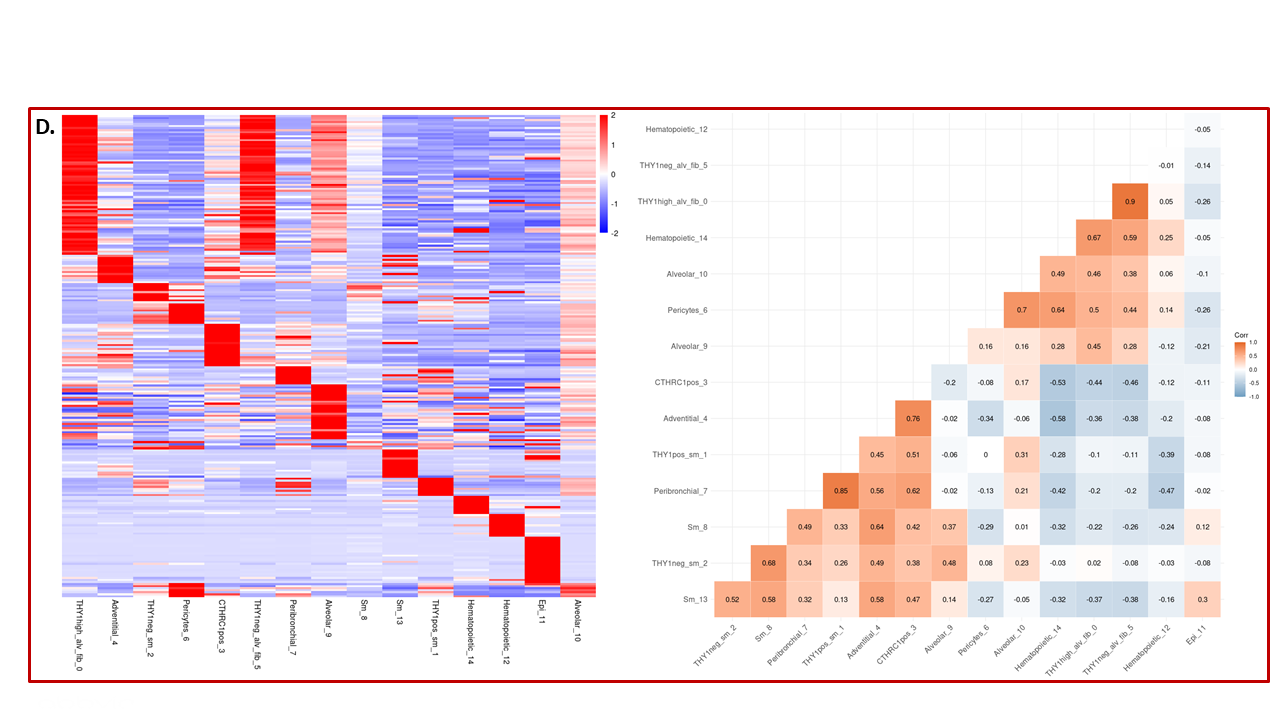

Supplement: S3 Fig — Clustering was performed using R package Seurat and cell types were identified using known markers. A. Total lung cell suspension. SPP1_monocytes_0: SPP1+ monocytes; Infl_monocytes_1: Inflammatory monocytes; ACKR1pos_endo_2: ACKR1+ endothelial cells; ACKR1neg_endo_3: ACKR1- endothelial cells; Fibroblasts_4: Fibroblasts; AT2_5 and AT2_23: Alveolar epithelial cell type II subpopulations; Th_6: helper T cells; Pericytes_7 and Pericytes_22: Pericyte subpopulations; HLAhigh_mac_8 and HLAhigh_mac_10: HLA class II high macrophage subpopulations; Sm_9: smooth muscle cells; Bcells_11 and Bcells_21: B cell subpopulations; Tc_12: cytotoxic T cells; AT1_13: Alveolar epithelial cell type I; PC_14: Plasma cells; Endo_15 and Endo_24: endothelial cell subpopulations; Ciliated_16: ciliated epithelial cells; Monocytes_17 and Monocytes_18: Monocyte subpopulations. B. Lineage sorted cells. THY1high_alv_fib_0: THY1 high alveolar fibroblasts; THY1pos_sm_1: THY1+ smooth muscle; THY1neg_sm_2: THY1- smooth muscle; CTHRC1pos_3: CTHRC1+ fibroblasts; Adventitial_4: Adventitial fibroblasts; THY1neg_alv_fib_5: THY1- alveolar fibroblasts; Pericytes_6: Pericytes; Peribronchial_7: Peribronchial fibroblasts; Sm_8 and Sm_13: smooth muscle cell subpopulations; Alveolar_9 and Alveolar_10: Alveolar fibroblast subpopulations; Epi_11: Epithelial cells; Hematopoietic_12 and Hematopoietic_14: Hematopoietic cells. C. Heatmap (left panel) and correlation matrix (right panel) in GSE47460 of genes included in the signature derived from the ‘Total lung cell suspension’ (shown in panel A) dataset across each cluster shown in panel A. D. Heatmap (left panel) and correlation matrix (right panel) in GSE47460 of genes included in the signature derived from the ‘Lineage sorted’ (shown in panel B) dataset across each cluster shown in panel B. (ZIP) [file pone.0248889.s003.zip › S3D_Fig.tif]

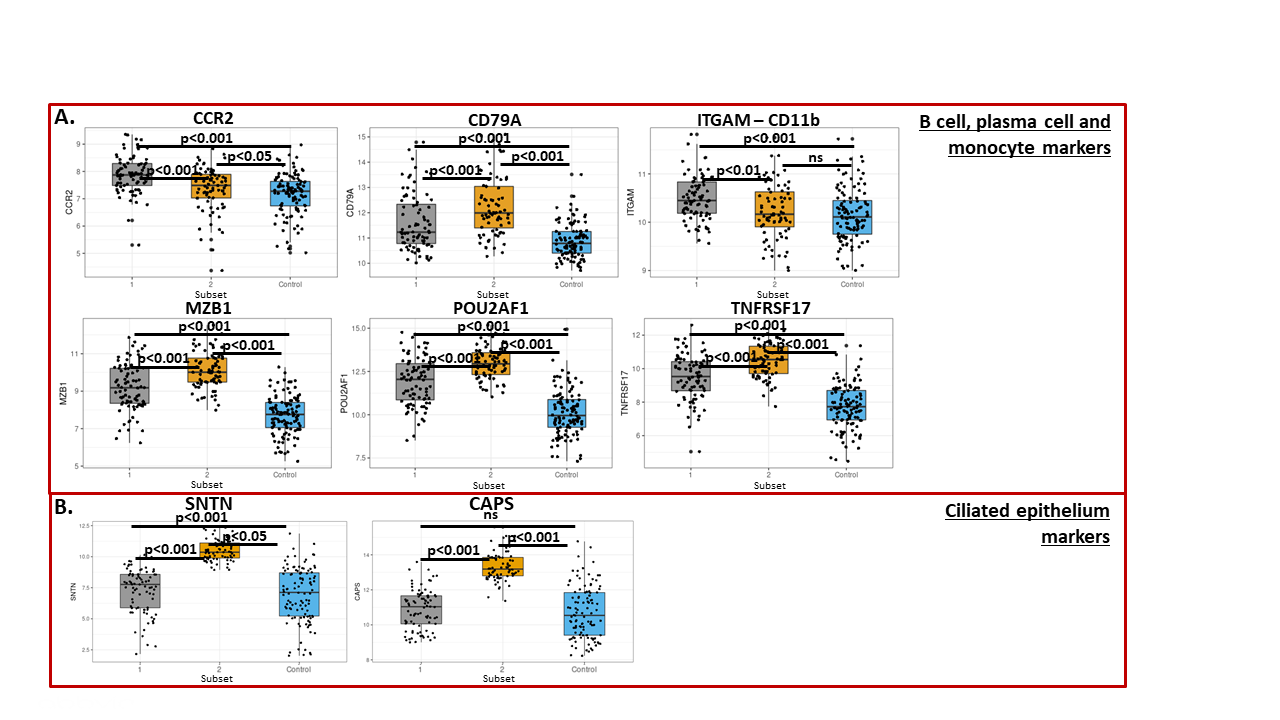

Supplement: S4 Fig — A. Expression of various B cell, plasma cell and myeloid markers in GSE47460 (Kaminski-LGRC bulk expression cohort) [14–17] subsets. B. Expression of ciliated epithelium cell markers in GSE47460 (Kaminski-LGRC bulk expression cohort) [14–17] subsets. Adjusted p values are reported on plots. (TIF) [file pone.0248889.s004.tif]

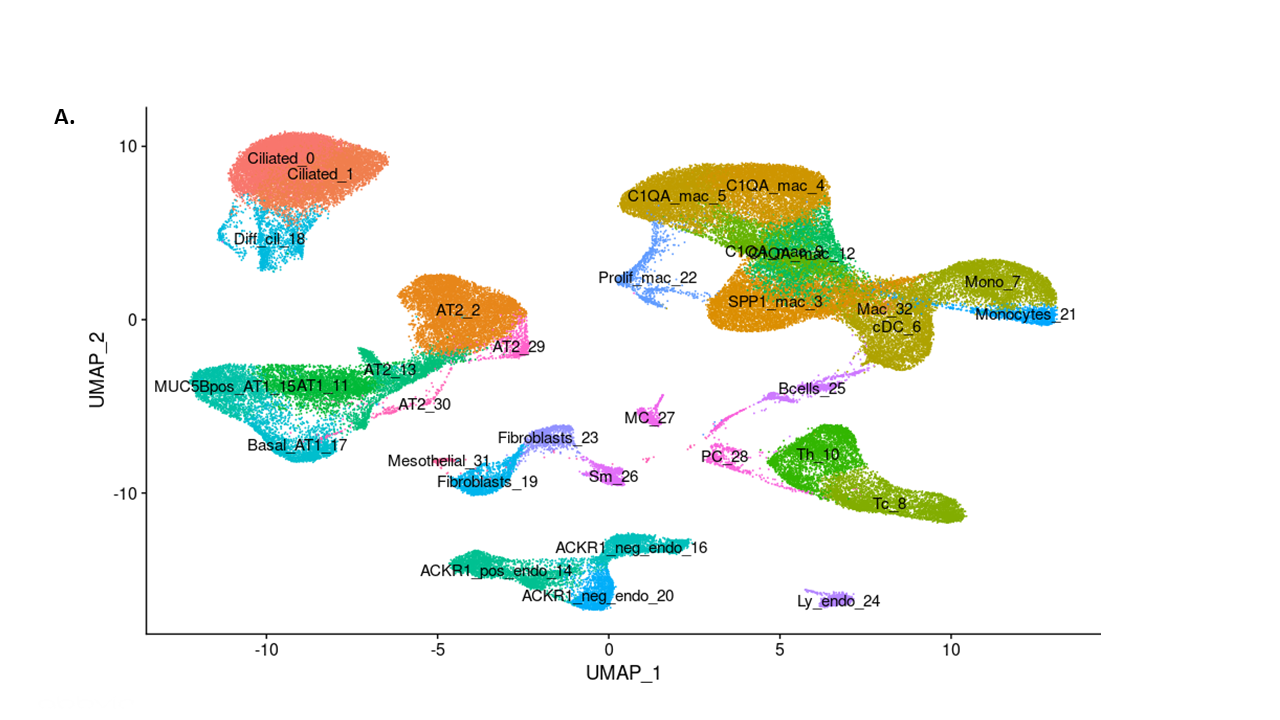

Supplement: S5 Fig — A. Cell type labels used based on re-analysis of IPF and healthy control data from GSE135893 (Kropski-Vanderbilt Univ single cell cohort) [24]. Clustering was performed using R package Seurat and cell types were identified using known markers. Ciliated_0 and Ciliated_1: Ciliated epithelial cell subpopulations; AT2_2, AT2_13, AT2_29, AT2_30: Alveolar epithelial cell type II subpopulations; SPP1_mac_3: SPP1+ monocytes/macrophages; C1QA_mac_4, C1QA_mac_5, C1QA_mac_9, C1QA_mac_12: C1QA+ macrophage subpopulations; Mono_7, Mono_21: Monocyte subpopulations; Tc_8: cytotoxic T cells; Th_10: helper T cells; AT1_11, MUC5Bpos_AT1_15, Basal_AT1_17: Alveolar epithelial cell type I subpopulations; ACKR1_pos_endo_14: ACKR1+ endothelial cells; ACKR1_neg_endo_16 and ACKR1_neg_endo_20: ACKR1- endothelial cell subpopulations; Diff_cil_18: Differentiating ciliated epithelial cells; Fibroblasts_19 and Fibroblasts_23: Fibroblast subpopulations; Sm_26: smooth muscle; Prolif_mac_22: Proliferating macrophages; Ly_endo_24: Lymphatic endothelium; Bcells_25: B cells; PC_28: Plasma cells; MC_27: mast cells; Mesothelial_31: mesothelial cells. B. Heatmap (left panel) and correlation matrix (right panel) in GSE47460 (Kaminski-LGRC bulk expression cohort) of genes included in the signature derived from the dataset shown in panel A. (ZIP) [file pone.0248889.s005.zip › S5A_Fig.tif]

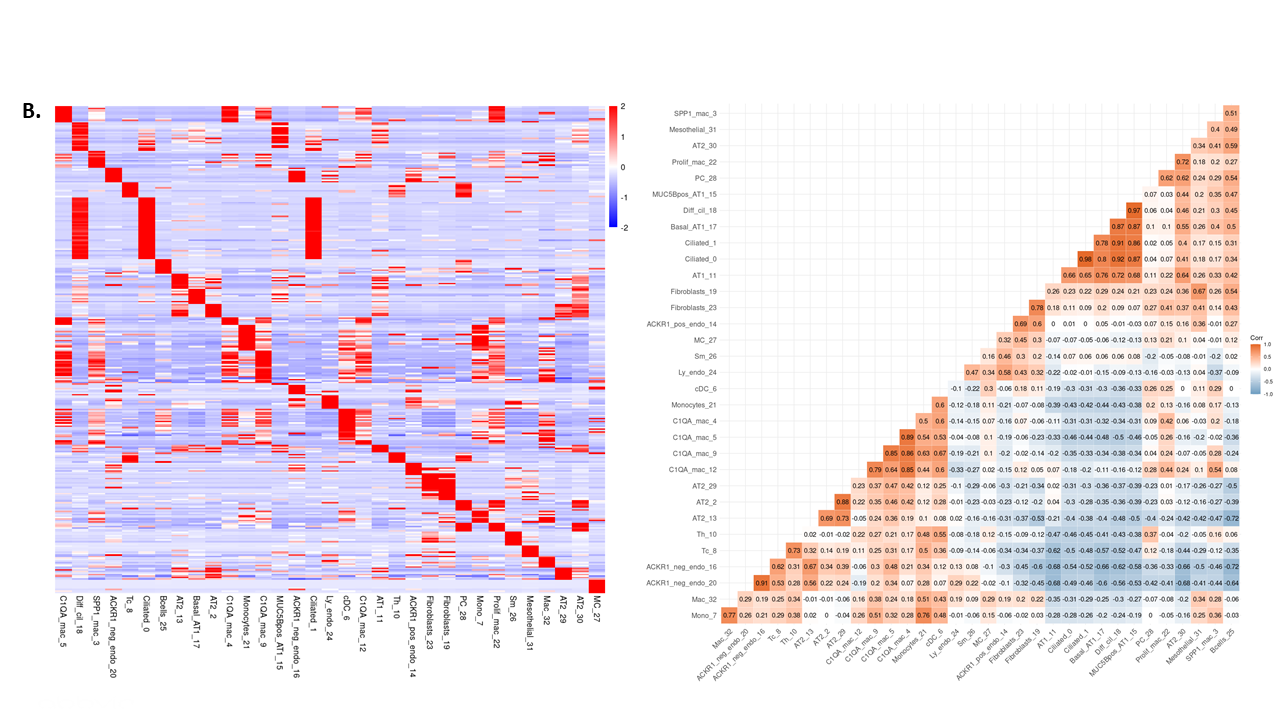

Supplement: S5 Fig — A. Cell type labels used based on re-analysis of IPF and healthy control data from GSE135893 (Kropski-Vanderbilt Univ single cell cohort) [24]. Clustering was performed using R package Seurat and cell types were identified using known markers. Ciliated_0 and Ciliated_1: Ciliated epithelial cell subpopulations; AT2_2, AT2_13, AT2_29, AT2_30: Alveolar epithelial cell type II subpopulations; SPP1_mac_3: SPP1+ monocytes/macrophages; C1QA_mac_4, C1QA_mac_5, C1QA_mac_9, C1QA_mac_12: C1QA+ macrophage subpopulations; Mono_7, Mono_21: Monocyte subpopulations; Tc_8: cytotoxic T cells; Th_10: helper T cells; AT1_11, MUC5Bpos_AT1_15, Basal_AT1_17: Alveolar epithelial cell type I subpopulations; ACKR1_pos_endo_14: ACKR1+ endothelial cells; ACKR1_neg_endo_16 and ACKR1_neg_endo_20: ACKR1- endothelial cell subpopulations; Diff_cil_18: Differentiating ciliated epithelial cells; Fibroblasts_19 and Fibroblasts_23: Fibroblast subpopulations; Sm_26: smooth muscle; Prolif_mac_22: Proliferating macrophages; Ly_endo_24: Lymphatic endothelium; Bcells_25: B cells; PC_28: Plasma cells; MC_27: mast cells; Mesothelial_31: mesothelial cells. B. Heatmap (left panel) and correlation matrix (right panel) in GSE47460 (Kaminski-LGRC bulk expression cohort) of genes included in the signature derived from the dataset shown in panel A. (ZIP) [file pone.0248889.s005.zip › S5B_Fig.tif]

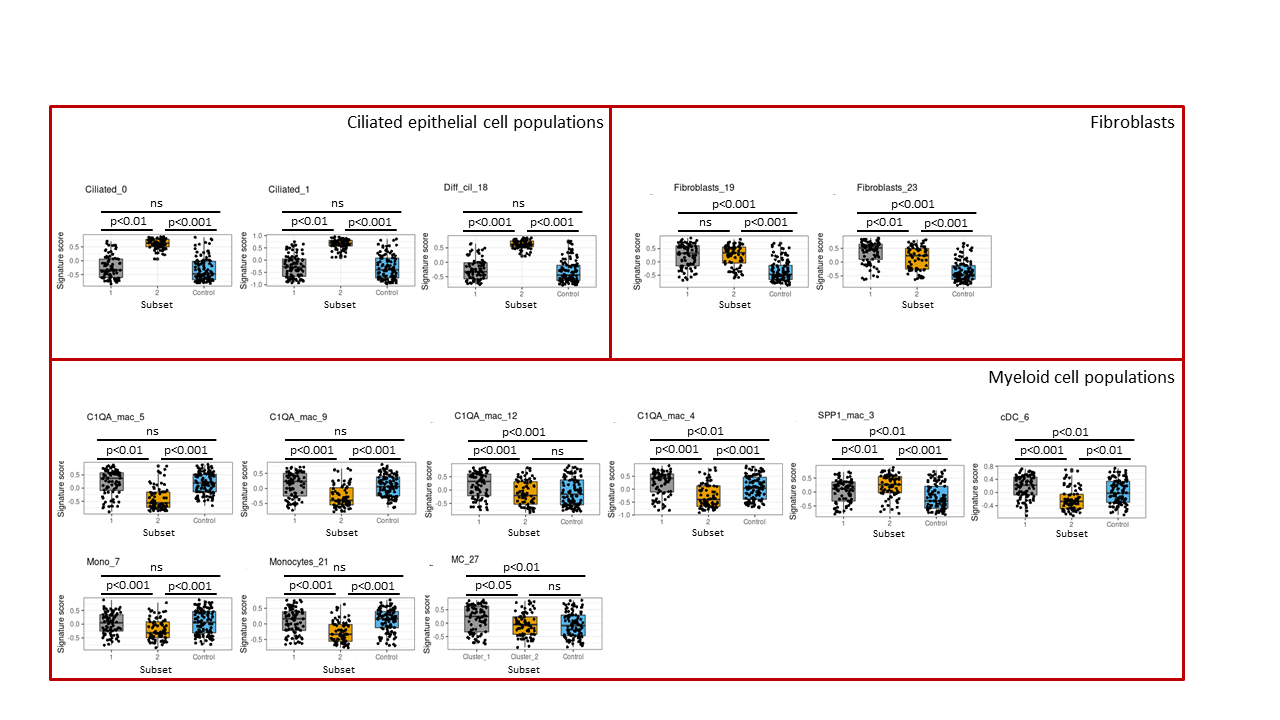

Supplement: S6 Fig — Only cell types with relevance to subsetting are shown. Nomenclature of cell types follows S5 Fig. (TIF) [file pone.0248889.s006.tif]

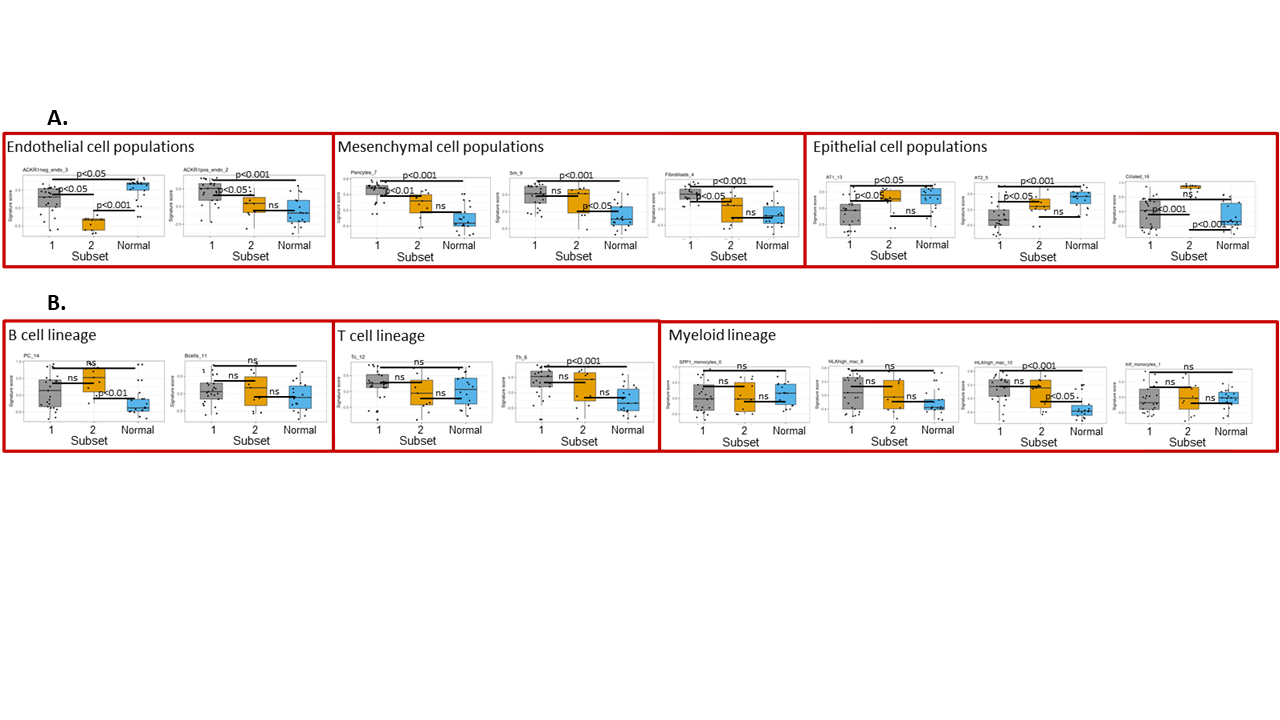

Supplement: S7 Fig — Only cell types with relevance to subsetting shown. Nomenclature of cell types follows S3 Fig. A. Non-hematopoietic populations from S3A Fig. B. Hematopoietic populations from S3A Fig. C. Cell populations from S3B Fig. (ZIP) [file pone.0248889.s007.zip › S7A-B_Fig.tif]

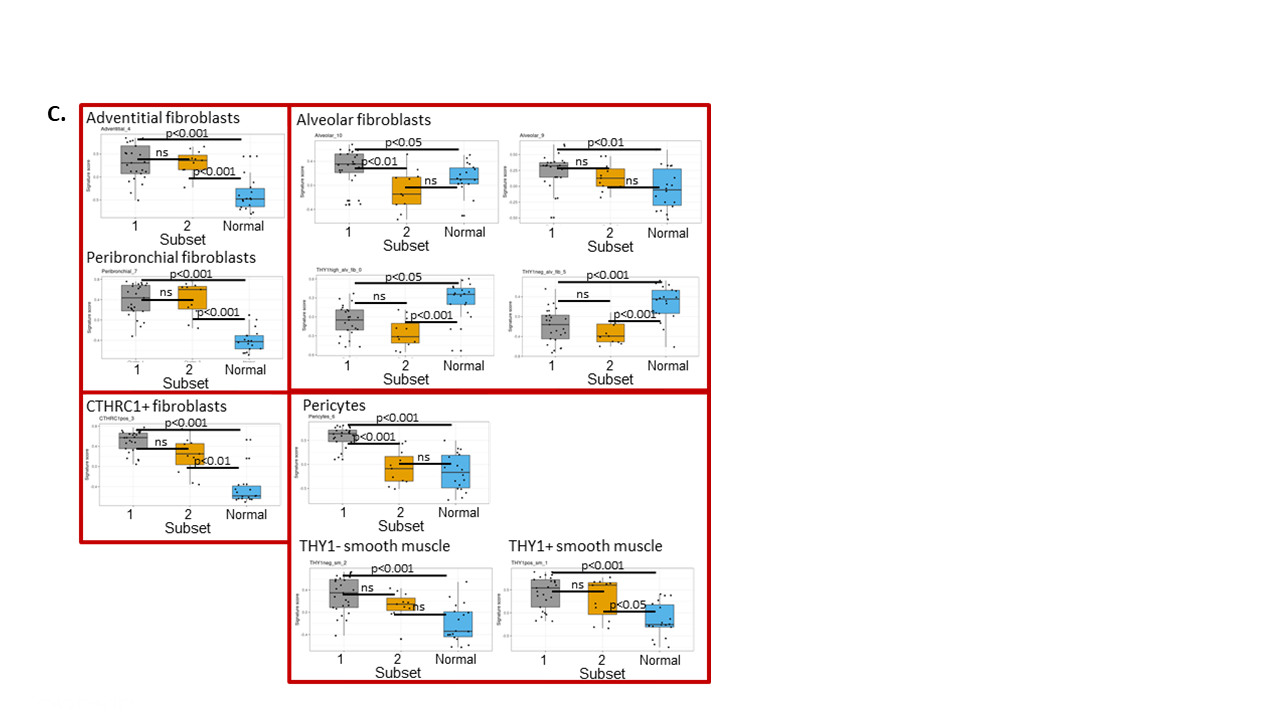

Supplement: S7 Fig — Only cell types with relevance to subsetting shown. Nomenclature of cell types follows S3 Fig. A. Non-hematopoietic populations from S3A Fig. B. Hematopoietic populations from S3A Fig. C. Cell populations from S3B Fig. (ZIP) [file pone.0248889.s007.zip › S7C_Fig.tif]

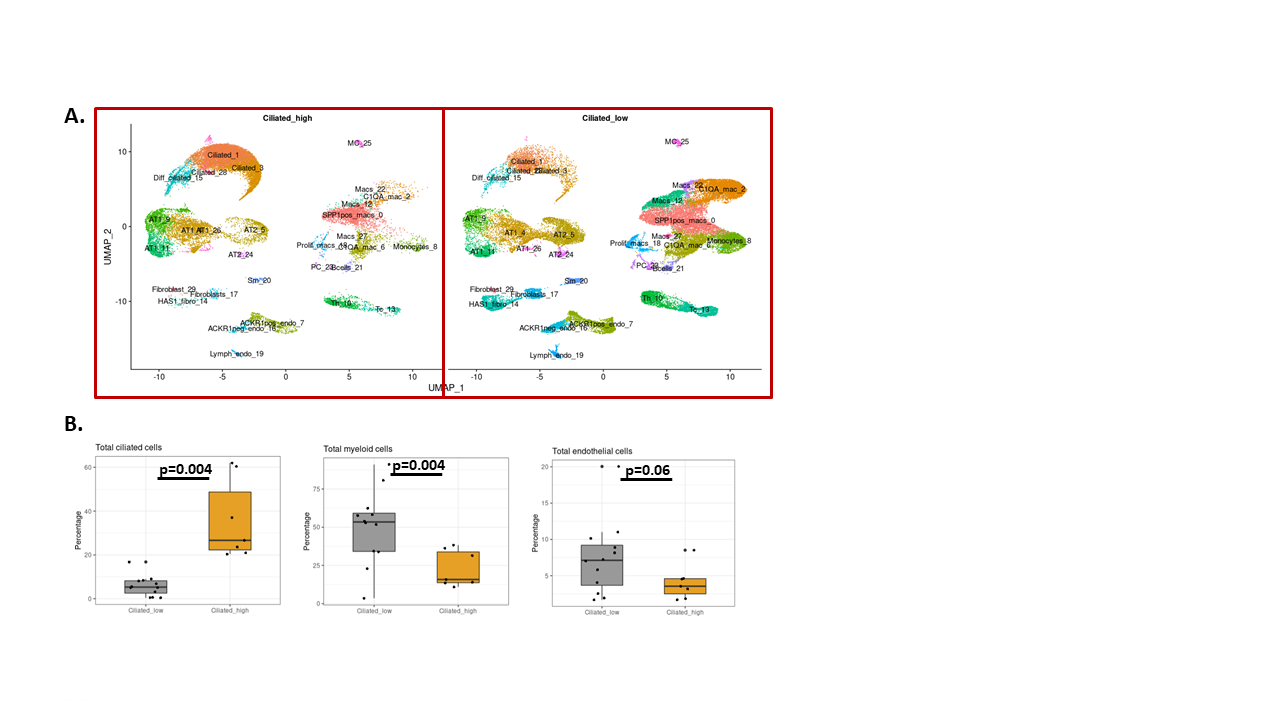

Supplement: S8 Fig — A. IPF samples in GSE135893 (Kropski-Vanderbilt Univ single cell cohort) [24] divided by the % of total ciliated cells in the data as shown in Fig 7A. SPP1pos_macs_0: SPP1+ monocytes/macrophages; Ciliated_1, Ciliated_3 and Ciliated_28: Ciliated epithelial cell subpopulations; C1QA_mac_2 and C1QA_mac_6: C1QA positive macrophage subpopulations; AT1_4, AT1_9, AT1_11, AT1_26: Alveolar epithelial cell type I subpopulations; AT2_5 and AT2_24: Alveolar epithelial cell type II subpopulations; ACKR1pos_endo_7: ACKR1+ endothelial cells; Monocytes_8: monocytes; Th10: helper T cells; Macs_12, Macs_22 and Macs_27: Macrophage subpopulations; Tc_13: cytotoxic T cells; HAS1_fibro_14: HAS1 positive fibroblasts; Diff_ciliated_15: differentiating ciliated epithelial cells; ACKR1neg_endo_16: ACKR1- endothelial cells; Fibroblasts_17 and Fibroblasts_29: Fibroblast subpopulations; Prolif_macs_18: Proliferating macrophages; Ly_endo_19: Lymphatic endothelium; Sm_20: smooth muscle; Bcells_21: B cells; PC_23: Plasma cells; MC_25: Mast cells. B. Differences in the percentage of Ciliated cells, Total myeloid cells and Endothelial cells between ‘Ciliated_low’ and ‘Ciliated_high’ subsets in GSE135893 (Kropski-Vanderbilt Univ single cell cohort) [24]. Percentages were calculated using cell numbers of the cell type indicated divided by the total number of cells in the data (subset based on Ciliated epithelial cells). Adjusted p values are reported on plots. (TIF) [file pone.0248889.s008.tif]

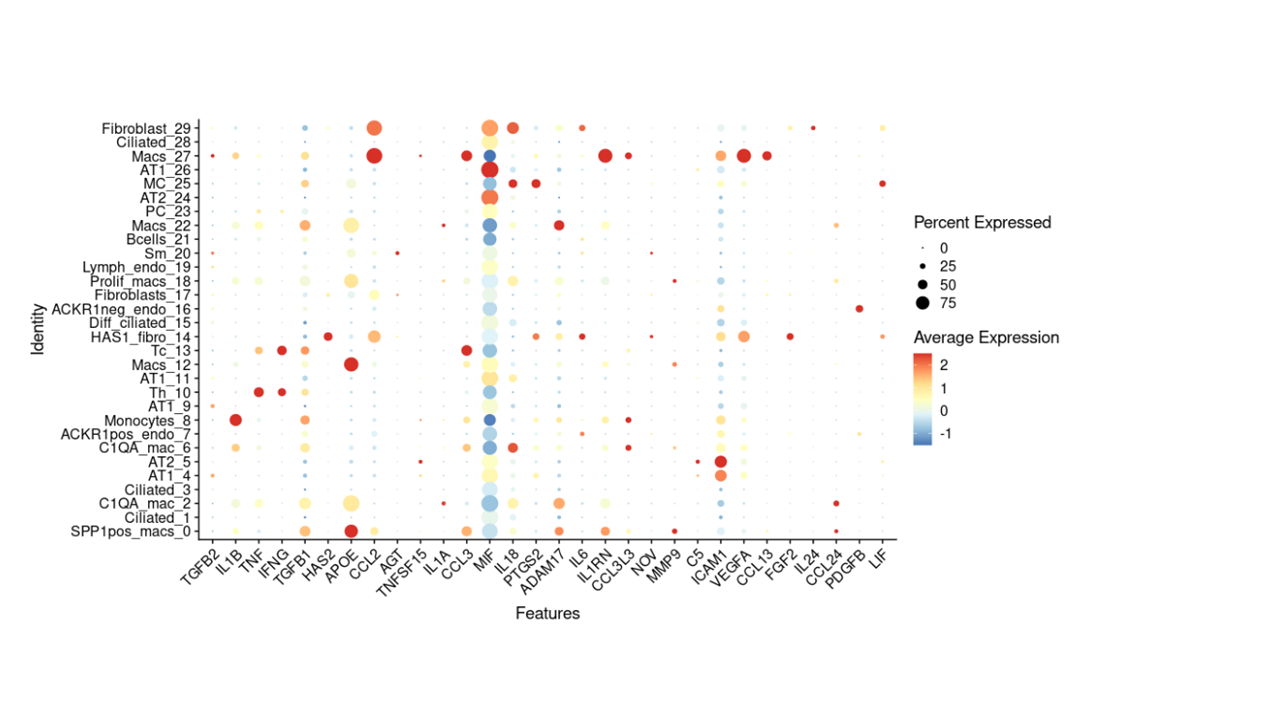

Supplement: S9 Fig — Size of circle indicated percent of cells gene on x axis is expressed in; color represents relative expression level. Nomenclature of cell clusters follows S8 Fig. (TIF) [file pone.0248889.s009.tif]
